# Host social organization and mating system shape parasite transmission opportunities in three European bat species

**DOI:** 10.1007/s00436-016-5323-8

**Published:** 2016-11-18

**Authors:** J. van Schaik, G. Kerth

**Affiliations:** 1 Department of Behavioural Ecology and Evolutionary Genetics, Max Planck Institute for Ornithology, Eberhard-Gwinner-Strasse, 82319 Seewiesen, Germany; 2 Zoological Institute & Museum, Greifswald University, J.-S.-Bach-Str. 11/12, D-17489 Greifswald, Germany

**Keywords:** *Myotis*, *Spinturnix*, Mite, Social system, Horizontal transmission

## Abstract

For non-mobile parasites living on social hosts, infection dynamics are strongly influenced by host life history and social system. We explore the impact of host social systems on parasite population dynamics by comparing the infection intensity and transmission opportunities of three mite species of the genus *Spinturnix* across their three European bat hosts (*Myotis daubentonii*, *Myotis myotis*, *Myotis nattereri*) during the bats’ autumn mating season. Mites mainly reproduce in host maternity colonies in summer, but as these colonies are closed, opportunities for inter-colony transmission are limited to host interactions during the autumn mating season. The three investigated hosts differ considerably in their social system, most notably in maternity colony size, mating system, and degree of male summer aggregation. We observed marked differences in parasite infection during the autumn mating period between the species, closely mirroring the predictions made based on the social systems of the hosts. Increased host aggregation sizes in summer yielded higher overall parasite prevalence and intensity, both in male and female hosts. Moreover, parasite levels in male hosts differentially increased throughout the autumn mating season in concordance with the degree of contact with female hosts afforded by the different mating systems of the hosts. Critically, the observed host-specific differences have important consequences for parasite population structure and will thus affect the coevolutionary dynamics between the interacting species. Therefore, in order to accurately characterize host-parasite dynamics in hosts with complex social systems, a holistic approach that investigates parasite infection and transmission across all periods is warranted.

**Electronic supplementary material:**

The online version of this article (doi:10.1007/s00436-016-5323-8) contains supplementary material, which is available to authorized users.

## Introduction

Host-parasite interactions are omnipresent in biological communities (Schmid-Hempel 2011). The epidemiology and population dynamics of parasites are influenced by many abiotic and biotic factors, especially the interaction of host and parasite life histories (Barrett et al. 2008; Nadler 1995). For example, in non-mobile permanent parasites that spend their whole life cycle on their hosts, infection intensity and diversity increases with increasing host group size (Côté and Poulin 1995; Patterson and Ruckstuhl 2013) and host density (Krasnov et al. 2002). Moreover, in parasites that are unable to disperse independently of their host, horizontal transmission is dependent on host spatiotemporal dynamics (Nunn and Altizer 2006). As a result, in many permanent parasites, infections are biased towards one host sex and show strong seasonal fluctuations as a function of the dynamics of host social interactions (Altizer et al. 2006; Krasnov et al. 2005).

The degree of parasite dispersal between host social groups will have strong consequences for the evolutionary dynamics between host and parasite. For example, genetic analyses of parasites on several bird species showed that parasite populations are largely undifferentiated among breeding grounds as a result of inter-colony contact and parasite transmission at communal wintering grounds or extensive prospecting by juvenile host individuals (Gomez-Diaz et al. 2012; Levin and Parker 2013). Parasite transmission between host populations are especially relevant from an evolutionary perspective, as the potential for parasite local adaptation is largely determined by the relative rate of gene flow in host and parasite (Gandon and Michalakis 2002), and the effective population size of both (Gandon and Nuismer 2009). Thus, when assessing host-parasite coevolutionary dynamics, it is essential to not only characterize the phases of the host’s annual cycle in which parasite infection levels are highest but also parasite phenology and transmission opportunities throughout the rest of the annual cycle. The latter, however, has rarely been done so far.

European temperate zone bats offer an interesting system for investigating the effects of host social system and seasonality on the prevalence, intensity, and transmission of permanent parasites. During summer, female bats typically form large, dense maternity colonies to raise their offspring (Kerth 2008). In addition to the large aggregation size, both females and juveniles show reduced immunocompetence and grooming compared to other times of the year (Christe et al. 2000). These conditions result in high levels of parasite infection intensity and parasite population growth in many bat colonies (Lourenço and Palmeirim 2008; Lučan 2006). In contrast to female bats, males are often solitary throughout the summer, although they may join female colonies or form male bachelor groups in some species (Safi and Kerth 2007). As a result, unlike most other mammalian systems (Schalk and Forbes 1997; Zuk and McKean 1996), females of most temperate zone bats have higher levels of parasite infection than males (e.g., Reckardt and Kerth 2009). Contact between the philopatric, closed female maternity colonies, and between females and males, is very rare during summer (Burland and Wilmer 2001; Kerth and van Schaik 2012). This results in limited horizontal transmission (used here as parasite exchange between different host colonies) opportunities for contact-transmitted parasites during this time (Bruyndonckx et al. 2009b; van Schaik et al. 2015).

In late summer and autumn, female maternity colonies disband and bats meet to mate, thereby also allowing for parasite transmission. Mating in temperate zone bat species is promiscuous and predominantly occurs either in temporary harems (Zahn and Dippel 1997), locally with males in the summer home range of the females (Senior et al. 2005), or during a behavior known as swarming, in which bats show intense flight activity at the entrance to underground sites used for hibernation in winter (Fenton 1969). During these encounters, parasites may be directly transmitted between females (in harems) or by males acting as a vector for parasite transmission between females from different colonies (van Schaik et al. 2014). In winter, bats hibernate either solitarily or in clusters (Dietz et al. 2007). In the latter case, additional parasite transmission may take place during this time. However, the reduced temperatures and torpor of the hosts correspondingly also reduces the metabolism and activity of ectothermic parasites (Christe et al. 2007). Taken together, although parasite populations are largest during summer, effective transmission and subsequent survival throughout autumn and possibly winter is key to parasite persistence and dispersal within the host meta-population. However, comparative studies of parasite population dynamics and transmission on bats with different social organization and mating systems in autumn are lacking.

In this context, we explore the transmission and temporal infection dynamics of a common genus of ectoparasite (wing mites of the genus *Spinturnix*) on three common European bat species at swarming sites during the autumn mating period. The three bat species, Daubenton’s bat (*Myotis daubentonii*), the greater mouse-eared bat (*Myotis myotis*), and Natterer’s bat (*Myotis nattereri*), all follow the same annual cycle described above, but differ substantially in social system, as summarized in Table 1. Most notably, they differ in social organization during summer (female maternity colony size and male bachelor groups) and mating system (temporary harem formation and promiscuous mating at swarming sites), providing a comparative framework to explore the effects of these host characteristics on parasite prevalence and distribution across the host sexes.

**Table 1:** Overview of the differences in host social system and their predicted effects on parasite transmission

	*Myotis daubentonii*	*Myotis myotis*	*Myotis nattereri*	Predicted effect
Summer (females)				
Social organization	Small colonies (10–100)^c^	Large colonies (50–2000)^c^	Small colonies (10–100)^c^	Larger colony size will yield higher parasite prevalence and intensity
Parasite prevalence/mean intensity	100%/10.3 ± 1.1^a,d^	100%/17.9 ± 1.22^e^	–	
Summer (males)				
Social organization	Solitary or male bachelor groups^b^	Solitary^b^	Solitary^b^	Male groups will yield higher parasite prevalence and intensity
Autumn				
Mating system	Swarming and Local mating^c, f^	Swarming and temporary harems^c, g^	Swarming^c^	Temporary harems will yield higher parasite transmission than swarming

^a^Samples were taken from lactating females only

^b^For all species, male bats are sporadically also observed in female maternity colonies

^c^Krapp (2004)

^d^Lučan (2006)

^e^Postawa and Szubert-Kruszyńska (2014)

^f^Encarnação and Reiners (2012)

^g^Zahn and Dippel (1997)

Wing mites of the genus *Spinturnix* infect almost all European bat species and show a clear coevolutionary pattern with their bat hosts (Bruyndonckx et al. 2009a). They are hematophagous and live exclusively on the wing and tail membranes of their host (Evans 1968). Larval stages develop inside the female mite, which gives birth to protonymphs that are able to move and feed independently (Evans 1968). Thus, mites never have to leave the host. Reproduction occurs almost exclusively in summer maternity colonies, with strong peaks in the presence of gravid female mites during host pregnancy and lactation, and no gravid females observed at low temperatures in winter (Lourenço and Palmeirim 2008). Mite infection has been observed to decrease during autumn and winter in *M. daubentonii* (Lučan 2006), although this pattern was not observed in autumn in the North American bat *Myotis lucifugus* (Webber et al. 2015). This reduction is presumably due to a combination of reduced mite reproduction and increased grooming activity and immunocompentence in their hosts. Mites are able to distinguish between host species (Giorgi et al. 2004) and host sex (Christe et al. 2007) and show a preference for juvenile hosts in summer maternity colonies (Christe et al. 2000). Mite infection increases host grooming activity and metabolism (Giorgi et al. 2001), and thus may impose a substantial cost, especially to juvenile hosts in maternity colonies (Lourenço and Palmeirim 2007). A study of mite population genetic structure across maternity colonies in *M. myotis* and *Myotis bechsteinii*, the latter having a similar social organization as *M. nattereri* (Kerth and van Schaik 2012; Rivers et al. 2005), found contrasting levels of genetic diversity and genetic differentiation between the maternity colonies of the two host species (van Schaik et al. 2014). Nevertheless, in both *M. myotis* and *M. bechsteinii*, substantial horizontal transmission of mites during the autumn mating season was implicated but could not be examined.

In the present study, we investigated mite infection of bats captured at three swarming sites in Germany throughout the bats’ autumn mating season. As mite infection intensity reduces rapidly after the disbandment of summer maternity colonies in autumn, and as there is substantial contact between conspecifics in the bats during mating at this time, we expect most successful mite horizontal transmission to occur during this period. Using linear mixed-effect models, we investigated the temporal dynamics of infection throughout the mating season in each of the different host age and sex classes. By comparing the results observed in each species, we aim to explore the effect of differences in host social system on parasite infection and opportunities for transmission. We hypothesize that differences in host female social organization will affect overall mite prevalence and intensity according to their summer maternity colony size, but that overall infection will decrease throughout the season in all species. Additionally, we predict that differences in host mating system and male social organization will affect the temporal dynamics of infection across host sexes during the autumn swarming season, with higher levels of mite prevalence and intensity in males of *M. daubentonii* as a result of their summer bachelor groups; moderate levels in *M. myotis* through extended contact with females in temporary harems; and low levels in *M. nattereri* through exclusive contact to females at swarming sites.

## Materials and methods

### Study site and bat capture

The study was carried out at three large swarming sites in Germany (Table 2), during August and September in 2011 and 2012. Bats were caught using mist nets (Schönsteinhöhle and Esperhöhle) or a harp trap (Brunnen Meyer) at the entrance of each site. In both years, each site was sampled four times (2011—26 August to 29 September; 2012—4 August to 27 September), approximately every 7 (2011) to 14 (2012) days, depending on weather conditions. This temporal range covers the peak swarming activity for all three species (Piksa et al. 2011).

**Table 2:** Overview of the three sampling locations, the number of individuals of each species caught at each site, and the total number of mites collected per species

Location	Coordinates	Year	Sampling events	*M. daubentonii*	*M. myotis*	*M. nattereri*
Brunnen Meyer	51.96, 7.37	2011	4	111	1	175
		2012	4	111	3	123
Esperhöhle	49.76, 11.29	2011	4	6	181	31
		2012	4	36	245	39
Schönsteinhöhle	49.81, 11.24	2011	4	14	52	252
		2012	4	38	43	137
Total			24	316	525	757
Total number of mites sampled				414	1873	241

For each individual bat, species, forearm length, mass, and age were recorded. Age was classified as either adult or young of year (YOY) based on the level of ossification of the epiphyseal joints (Brunet-Rossinni and Wilkinson 2009). At the Schönsteinhöhle and Esperhöhle, all captured bats were temporarily marked on the thumbnail using a permanent marker to exclude recaptured individuals. At the Brunnen Meyer, *M. nattereri* and *M. daubentonii* had been marked for a concurrent study (F. Meier, L. Grosche, unpublished data), thereby excluding recaptures. During our study, bats of ten other species were also caught (*n* = 214); a summary of these captures (including mite prevalence and intensity) is given in the Online Resource (Table S1). Notably, several species were caught in large numbers but were rarely or never infected with mites (see “Discussion” section).

Captured bats were placed in separate capture bags to avoid contamination of mites prior to sampling. Bat wing and tail membranes were systematically inspected and all mites were collected and stored in 96% alcohol.

### Data analysis

Infection dynamics were investigated using two standard measures of parasite infection: prevalence, the proportion of hosts infected with one or more mites; and intensity, the number of mites on a single infected host excluding hosts which are not parasitized (sensu Bush et al. 1997), using generalized linear mixed-effects models (package lme4 in R 2.14.1; Bates et al. 2014; R Development Core Team 2015). First, for each species and each measure (prevalence, intensity), we ran a separate model in which we compared overall infection levels across the four host age and sex combinations (adult male, adult female, YOY male, YOY female; henceforth referred to as host classes), and a second set of models comparing the overall temporal trend of infection pooled across all host classes. In these models, host class or capture date (respectively) were used as fixed effects, and capture site and year were included as random effects. Subsequently, to investigate the change in infection level of each host class throughout the swarming season, the models were run with both host class and capture date as fixed effects and capture site and year as random effects.

In all cases, mite prevalence was modeled using logit-linked binomial errors. Mite intensity was modeled using a Poisson distribution (log-link). As we intended to compare the temporal infection dynamics of male hosts to those of females, adult female hosts were used as the intercept for all models, and all other classes were evaluated relative to these estimates. To assess model output and to evaluate differences among host classes, we used the sim function (package arm; Gelman et al. 2014) to generate 95% credibility intervals based on 2000 simulations of the posterior distribution. Credibility intervals that did not include zero were considered to be significant in the frequentist sense. For all models, the influence of the random effects (sampling location and sampling year) is summarized in the Online Resource (Table S2).

As mite intensity (log-link) models were overdispersed, both with and without day as an additional fixed effect, they were also analyzed using a zero-truncated distribution (pospoisson: package VGAM; Yee and Wild 2010). In this model, host class was included as a fixed effect with no random effects included in the model. The additional models did not yield qualitatively different results (Online Resource, Table S3).

## Results

### Capture overview

A total of 1598 bats of the three target species were caught, and 2528 mites were collected from them (Table 2). We caught more male than female bats in all three species, although male bias was stronger in *M. daubentonii* and *M. nattereri* than in *M. myotis* (0.66, 0.69, and 0.56, respectively). Approximately one third of the bats caught were identified as young of year (0.30, 0.37, and 0.37, respectively).

### *Myotis daubentonii*

**Fig. 1:**
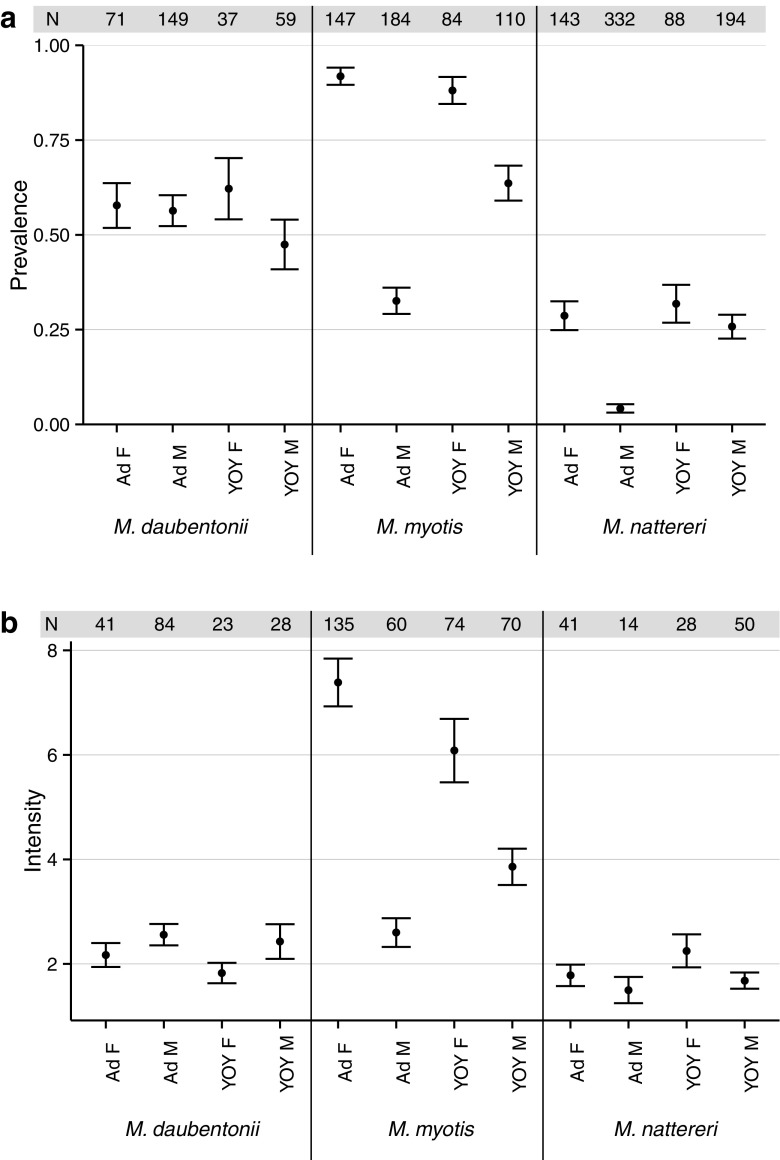
Overall **a** prevalence and **b** intensity of mites on bats captured (mean ± SE), subdivided by host class. *N* denotes the number of sampled individuals per host class

**Fig. 2:**
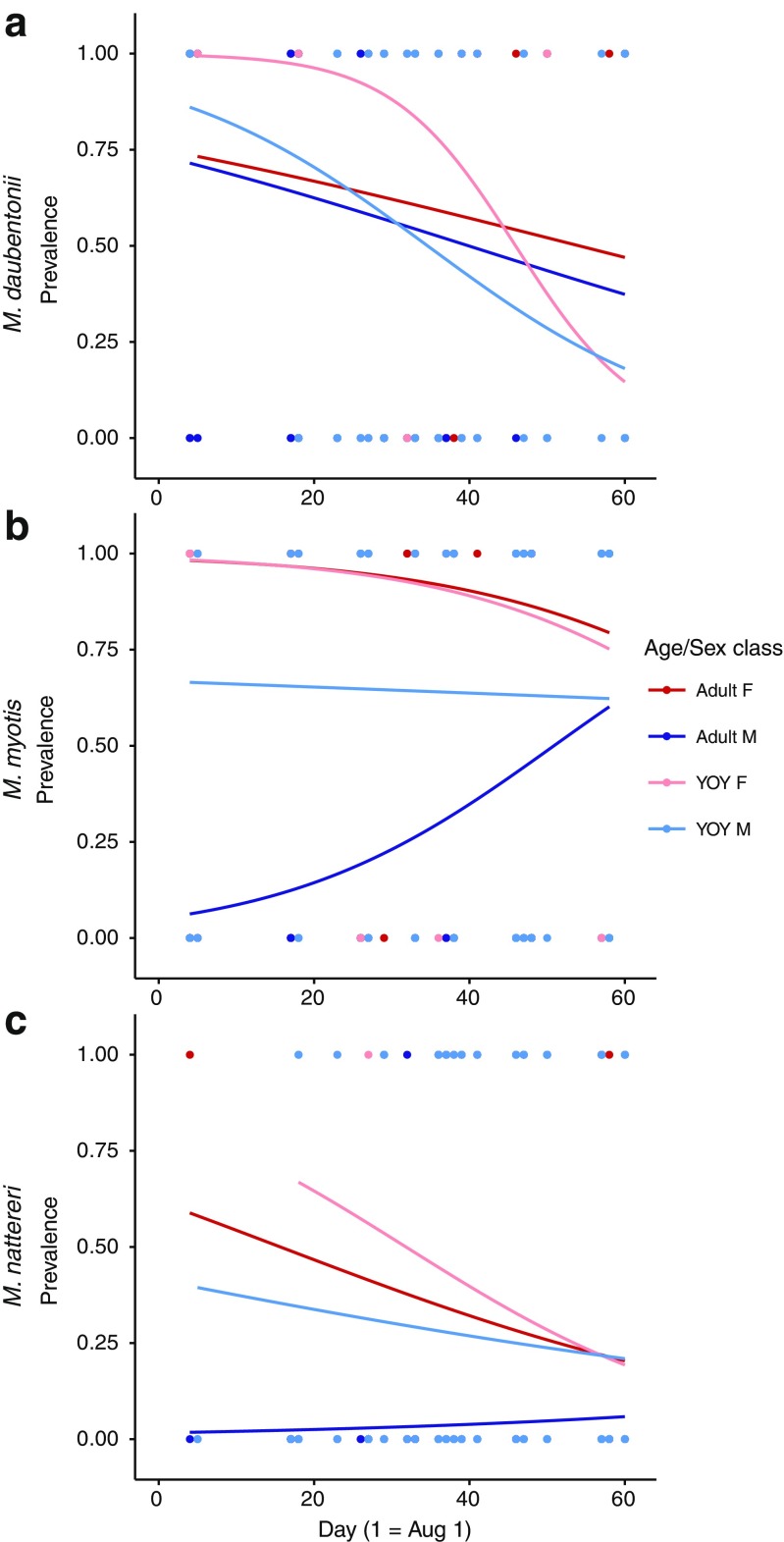
Temporal dynamics of mite prevalence during the autumn mating season in **a** *M. daubentonii*, **b** *M. myotis*, and **c** *M. nattereri*

**Fig. 3:**
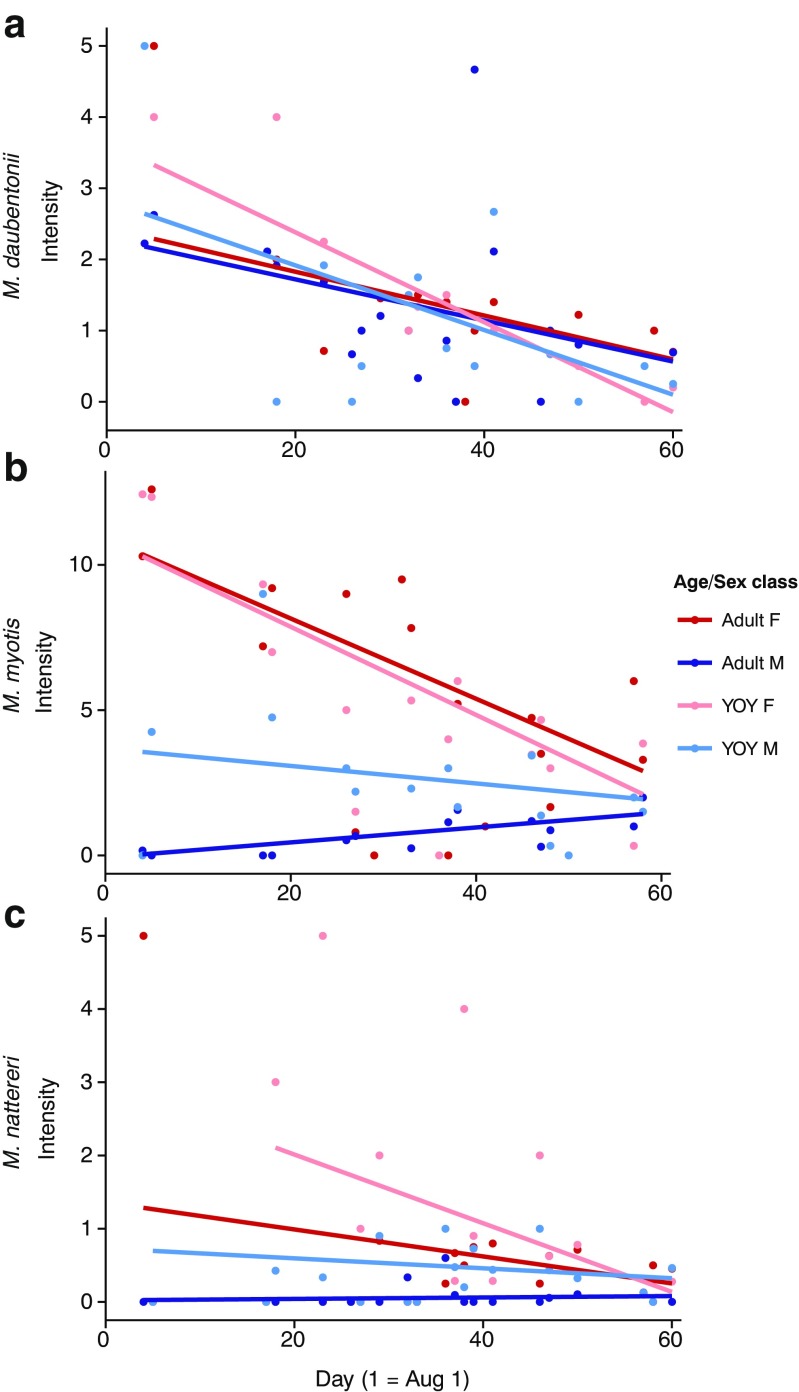
Temporal dynamics of mite intensity during the autumn mating season in **a** *M. daubentonii*, **b** *M. myotis*, and **c** *M. nattereri. Points* indicate average intensity per host class per sampling day

In *M. daubentonii*, overall prevalence and intensity did not differ between host classes (Table 3; Fig. 1). Pooled across host classes, mite prevalence and intensity all decreased over time during the swarming season (Table 3). The temporal decrease in both infection parameters was comparable across adult females, adult males, and YOY males (Table 4; Figs. 2a and 3a). However, a stronger decrease in mite prevalence was observed in YOY females relative to adult female hosts (Table 4).

**Table 3:** Estimated effect sizes and 95% credibility intervals for mite prevalence (binomial; logit-scale) and intensity (Poisson; log-scale) for each host class pooled across all capture dates (top) and the overall temporal trend pooled across all host classes (bottom) with capture location and year as random effects

		Prevalence		Intensity	
Species	Fixed effect	Estimate	95% CI	Estimate	95% CI
Host class					
*M. daubentonii*	Intercept (Ad F)	0.33	(−0.35, 0.99)	0.76	(0.56, 0.98)
	Ad M	−0.13	(−0.71, 0.42)	0.16	(−0.08, 0.41)
	YOY F	0.08	(−0.74, 0.87)	−0.17	(−0.54, 0.19)
	YOY M	−0.48	(−1.19, 0.22)	0.11	(−0.19, 0.43)
*M. myotis*	Intercept (Ad F)	2.42	(1.82, 3.04)	1.55	(0.73, 2.33)
	Ad M	−3.15^a^	(−3.82, −2.48)	−0.88^a^	(−1.05, −0.70)
	YOY F	−0.42	(−1.37, 0.38)	−0.14^a^	(−0.26, −0.03)
	YOY M	−1.86^a^	(−2.59, −1.15)	−0.57^a^	(−0.70, −0.44)
*M. nattereri*	Intercept (Ad F)	−0.91	(−1.33, −0.50)	0.58	(0.34, 0.80)
	Ad M	−2.18^a^	(−2.83, −1.54)	−0.17	(−0.66, 0.28)
	YOY F	0.1	(−0.46, 0.69)	0.23	(−0.11, 0.57)
	YOY M	−0.17	(−0.64, 0.32)	−0.06	(−0.37, 0.27)
Temporal					
*M. daubentonii*	Intercept (1 Aug)	1.39	(0.76, 2.03)	1.28	(1.06, 1.50)
	Day	−0.03^a^	(−0.05, −0.02)	−0.01^a^	(−0.02, −0.01)
*M. myotis*	Intercept (1 Aug)	0.52	(0.06, 0.96)	2.26	(2.03, 2.49)
	Day	0.00	(−0.01, 0.01)	−0.02^a^	(−0.02, −0.02)
*M. nattereri*	Intercept (1 Aug)	−1.08	(−1.90, −0.24)	1.13	(0.61, 1.64)
	Day	−0.01	(−0.03, 0.01)	−0.01^a^	(−0.02, 0.00)

^a^Denotes estimates where the 95% CI does not include zero

**Table 4:** Estimated effect sizes and 95% credibility intervals for mite prevalence (binomial; logit-scale) and intensity (Poisson; log-scale) of the temporal change in infection per host class, with capture location and year as random effects

		Prevalence		Intensity	
Species	Fixed effect	Estimate	95% CI	Estimate	95% CI
*M. daubentonii*	Intercept (Ad F × day)	−0.02	(−0.06, 0.02)	−0.01	(−0.03, 0.00)
	Ad M × day	−0.01	(−0.05, 0.04)	0.00	(−0.01, 0.02)
	YOY F × day	−0.11*	(−0.19, −0.02)	−0.01	(−0.04, 0.02)
	YOY M × day	−0.04	(−0.10, 0.02)	0.00	(−0.03, 0.02)
*M. myotis*	Intercept (Ad F × day)	−0.05	(−0.09, 0.01)	−0.02	(−0.02, −0.01)
	Ad M × day	0.11^a^	(0.06, 0.15)	0.02^a^	(0.00, 0.03)
	YOY F × day	−0.01	(−0.07, 0.06)	0.00	(−0.01, 0.00)
	YOY M × day	0.05	(−0.01, 0.09)	0.00	(0.00, 0.01)
*M. nattereri*	Intercept (Ad F × day)	−0.03	(−0.06, 0.01)	−0.01	(−0.03, 0.01)
	Ad M × day	0.05	(−0.01, 0.10)	0.01	(−0.05, 0.06)
	YOY F × day	−0.02	(−0.08, 0.03)	−0.01	(−0.04, 0.02)
	YOY M × day	0.01	(−0.03, 0.05)	0.01	(−0.02, 0.04)

^a^Indicates estimates where the 95% CI does not include zero

### *Myotis myotis*

In *M. myotis*, overall prevalence and intensity were all higher in adult females than in both adult male and YOY male hosts (Table 3, Fig. 1). Prevalence levels between adult females and YOY females did not differ, but mite intensity was lower in YOY females (Table 3). Pooled across host classes, mite prevalence did not change temporally, but mite intensity decreased throughout the swarming season (Table 3).

Temporal dynamics of mite prevalence and intensity did not differ between adult females and both male and female YOY, with decreases in both parameters throughout the mating season (Table 4; Figs. 2b and 3b). Conversely, mite prevalence and intensity increased over the swarming season in adult male bats (Table 4). Indeed, by the end of the swarming season, prevalence and intensity levels in adult male hosts approached those seen in the other host classes (Figs. 2b and 3b).

### *Myotis nattereri*

Overall prevalence and intensity in *M. nattereri* were low (Fig. 1) and did not differ between adult females and YOY of both sexes (Table 3). Mite prevalence on adult males was drastically lower (Table 3), but mite intensity did not differ relative to the levels found in adult females. Similar to *M. myotis*, when pooled across host classes, mite prevalence did not change temporally, but mite intensity decreased throughout the swarming season (Table 3).

Both prevalence and intensity decreased over the swarming season in adult females and both male and female YOY (Table 4, Figs. 2c and 3c), whereas they increased over time in adult male hosts (Figs. 2c and 3c), albeit insignificantly (Table 4).

## Discussion

Through a comparative framework across three European bat species and their parasitic wing mites, we observed substantial differences in initial parasite infection of all host classes and the infection dynamics of male hosts between species as predicted based on the differences in their social systems.

### Overall mite prevalence and intensity

In female and YOY hosts, overall prevalence and intensity levels were highest in *M. myotis*, intermediate in *M. daubentonii*, and lowest in *M. nattereri*. In male hosts, prevalence and intensity were highest in *M. daubentonii* with lower levels of infection in the other two species. The observed relative infection rates of the hosts closely mirror the size of host summer aggregations, where *M. myotis* have larger maternity colonies than the other two species, and males of *M. daubentonii* form bachelor group aggregations while the males of the other species are almost exclusively solitary during summer. Notably, although overall prevalence and intensity values of females also broadly correspond to the differences in body size between the species (*M. myotis* > *M. daubentonii* and *M. nattereri*), this pattern does not hold for the males of these species and across the other bat species also captured at the same sites (compare in Online Resource; Table S1). Additionally, body size does not account for the marked differences observed between sexes within species, nor the different temporal dynamics of mite infection observed in the three host species. Therefore, we conclude that host social organization in summer, particularly aggregation size, shapes the overall level of mite infection seen during the autumn mating phase across these species.

### Temporal dynamics

Despite the difference in overall mite prevalence and intensity across species, the overall temporal dynamics of mite infection of female and YOY hosts (i.e., all host classes living in maternity colonies previous to our sampling) were broadly similar between the investigated species. Overall mite prevalence and intensity levels at the beginning of the autumn mating season were already lower than peak infection levels observed in host summer colonies (Encarnação et al. 2012; Lučan 2006; Postawa and Szubert-Kruszyńska 2014). Additionally, mite intensity continued to decrease throughout the investigated period in all species, and in *M. daubentonii*, overall prevalence also decreased. Presumably, this decrease is the result of the disbandment of host maternity colonies during our sampling period, an increase in immunocompentence and grooming behavior of the hosts (Christe et al. 2000), and the cessation of reproduction in the mites (Deunff and Beaucournu 1981; Lourenço and Palmeirim 2008). Interestingly, in contrast to the decrease observed in this study, no decrease in parasite load was observed in *M. lucifugus* during the autumn mating season in Canada (Webber et al. 2015). This was suggested to be a result of the cold climate, which resulted in increased host aggregation during the mating season and a comparatively short mating season due to the earlier onset of hibernation (Webber et al. 2015), a pattern which is not observed in the three species studied here. Taken together, these results highlight the role of the abiotic environment in shaping host social organization and subsequently host-parasite dynamics.

In contrast to female and YOY hosts, male temporal infection dynamics did differ between species. In *M. myotis*, adult male bats were initially rarely infected, but by the end of the season, prevalence and intensity of mites reached similar infection levels to those seen in adult female and YOY hosts at this time, suggesting that substantial transmission is taking place during the autumn mating period. This transmission can either occur during contact between hosts at swarming sites or in the temporary harems formed during this period in which adult males roost together with several females (either simultaneously or consecutively) for one or several days (Zahn and Dippel 1997). In *M. nattereri*, mite prevalence and intensity on male bats was similarly low initially, but, unlike the increase seen in *M. myotis*, only trended upward throughout the autumn mating period. This is likely due to the lower overall prevalence and intensity observed across all host classes in *M. nattereri* combined with the limited transmission opportunities afforded by the short inter-individual contact during mating at swarming sites in contrast to the relatively long contact time in harem groups in *M. myotis*. Finally, in *M. daubentonii*, temporal infection dynamics of male bats did not differ from those seen in female and YOY hosts, as predicted from their aggregation in male bachelor groups throughout the summer. As all host classes showed similar prevalence and intensity levels throughout the season, we cannot infer that transmission of parasites is taking place as in the other bat species, although it appears likely given that the majority of individuals are infected and individuals must come into contact for mating. Altogether, across the three systems, we find that the temporal dynamics of mite infection strongly reflect the variation in host mating system as well as the changes in host social organization during this period.

### Host social system and parasite infection dynamics

The observed correlations between host social system and mite infection dynamics have several important implications for parasite population structure. In *M. myotis*, the formation of temporary harems during the mating season is particularly likely to increase parasite transmission, as individuals remain in contact for much longer than during the brief mating events at swarming sites. In addition to resulting in higher infection levels of male bats, *Spinturnix myoti* mites may also be transmitted directly from female to female possibly originating from different colonies in harems where multiple females roost with a male simultaneously (Zahn and Dippel 1997). Moreover, the high overall prevalence of mites on all host classes is likely to facilitate further mite transmission during winter. As a result, the mite meta-population should be well mixed and its effective population size is expected to be much larger than in the other investigated species. Support for this prediction is found in an analysis of mite population genetic structure across six *M. myotis* maternity colonies, which found high overall genetic diversity and very little genetic differentiation between mites from different *M. myotis* colonies (van Schaik et al. 2014).

In *M. daubentonii*, the presence of male bachelor groups increases the number of host clusters available to mites during their reproductive period (summer). This should increase the overall parasite persistence within the local meta-population, making regional extinctions (see *M. nattereri* discussion below) far less likely. Additionally, it effectively increases the amount of transmission of mites during the bats’ mating season, as mites no longer need to transfer exclusively to female hosts, but can additionally persist on male hosts. Therefore, despite lower overall levels of mite infection than in *M. myotis*, mite (genetic) population structure is likely to be similarly well mixed and temporally stable across the mite meta-population. Interestingly, mites have previously been shown to have a preference for female hosts over male hosts in *M. daubentonii* (Christe et al. 2007), but this preference was not reflected in the mite prevalence and intensities observed here. However, we did find a slightly higher prevalence and intensity of mites on adult hosts of both sexes over YOY hosts. This may indicate a preference for adult hosts of either sex during the mating (and hibernation) phase, which would be advantageous as these host individuals are more likely to survive than YOY (Lentini et al. 2015).

Finally, in *M. nattereri*, we observed low overall level of mite infection and limited transmission during the swarming season. In combination with the small female maternity colony size, we therefore anticipate survival of mite populations from year to year in individual host populations to be much lower than in the other investigated mite species. As a result, local extinction/re-colonization events are expected to be frequent (Reckardt and Kerth 2009). Such local extinctions in individual host summer maternity colonies would further lower the overall prevalence and intensity of mites at the onset of autumn swarming in the subsequent year. This may explain the observed difference in prevalence and intensity between *M. nattereri* and *M. daubentonii* despite their similar summer maternity colony sizes. Indeed, if the density of host colonies in a particular area is low, and local mites populations within several host maternity colonies go extinct, horizontal transmission of mites at swarming sites may be insufficient to prevent regional extinction of mites within the catchment area of those swarming sites.

A comparison with *Myotis bechsteinii*, which has a similar social system to *M. nattereri* (Dietz et al. 2007), shows that such a scenario may be plausible. In this study, we captured over 70 *M. bechsteinii*, but we did not find any to be infected with mites (see Table S1) despite the fact that such mites are common in several nearby (±100 km) German regions (Bruyndonckx et al. 2009b; van Schaik et al. 2014). This stochasticity in local extinction patterns can also have strong consequences for parasite population genetic structure in regions where the parasite does not go regionally extinct. In *M. bechsteinii*, mite populations from different maternity colonies were temporally unstable as a result of the limited transmission and low number of founding individuals per host colony (Bruyndonckx et al. 2009b; van Schaik et al. 2014).

## Conclusion

Taken together, our findings illustrate several important effects of host social system on parasite population dynamics. As shown in many other vertebrate systems, host group size and density during the reproductive period of both host and parasite strongly influence parasite infection intensity (e.g., Côté and Poulin 1995; Patterson and Ruckstuhl 2013; Rifkin et al. 2012). However, as a result of host social behavior restricting interactions between different host social units, horizontal transfer of parasites between such aggregations may be limited (Barrett et al. 2008). In hosts with strong seasonality, social structure may subsequently change drastically through the year, as is the case in temperate zone bats. These changes provide additional horizontal transmission opportunities for permanent ectoparasites. This could happen either via hosts of the opposite sex acting as vectors or through direct contact of conspecifics from different colonies as shown here for bats and in other studies for migratory bird species with communal wintering grounds (Gomez-Diaz et al. 2012). In addition, seasonal changes in the social organization of hosts are often coupled with changes in host behavior (e.g., grooming rate) or physiology (e.g., hibernation), which may also influence parasite survival. Therefore, both the social organization of hosts during the main reproductive season as well as the transmission opportunities and challenges to parasite survival during the “suboptimal” seasons will combine to affect parasite population and genetic structure.

Notably, the resulting parasite population structure will ultimately affect the coevolutionary dynamics between host and parasite (Boots et al. 2004; Gandon and Nuismer 2009; Lion and Boots 2010). Therefore, in order to understand both the proximate and ultimate dynamics of parasite infection in hosts where the social organization changes throughout the year, a holistic approach that includes infection during the main reproductive period, but also the focal points of parasite transmission and the critical phases for parasite survival, is required.

## Electronic supplementary materials

Below is the link to the electronic supplementary material.

ESM 1 Supplementary analyses and figures as referenced in the text. (PDF 101 kb)

ESM 2 Raw data supporting the findings presented in this manuscript. (PDF 1045 kb)
