# Supplementary material for: Host social organization and mating system shape parasite transmission opportunities in three European bat species
Source: Parasitol Res. 2016 Nov 18;116(2):589–99. doi: 10.1007/s00436-016-5323-8 (PMC5258804; doi:10.1007/s00436-016-5323-8)
Supplement: Supplementary file 1 — Supplementary analyses and figures as referenced in the text. (PDF 101 kb) [file 436_2016_5323_MOESM1_ESM.pdf]

Electronic Supplementary Material

van Schaik J[1§] & Kerth G[2] (2016) Host social organization and mating system shape parasite transmission opportunities in three European bat species.

[1] *Department of Behavioural Ecology and Evolutionary Genetics, Max Planck Institute for Ornithology, Eberhard-Gwinner-Strasse, 82319 Seewiesen, Germany*
[2] *Zoological Institute & Museum, Greifswald University, J.-S.-Bach-Str. 11 / 12, D-17489 Greifswald, Germany.*
[§] Corresponding author: Jaap van Schaik; jaapvanschaik@gmail.com; +49 8157 932 416

Table ESM1: Overview of the other bat species caught at swarming sites during this study, including their mite prevalence and intensities. Notably, mites were entirely absent on *M. bechsteinii* despite moderately high sample sizes.

Species	Sex - Age	N	$N_{infected}$	N_{paras}	Prevalence	Intensity
B. barbastellus	M Adult	35	9	17	0.26	1.88
	M YOY	3	1	2	0.33	2
	F Adult	7	3	3	0.43	1
E. nilssonii	M Adult	3	1	1	0.33	1
	M YOY	1	0	0	0	-
E. serotinus	M Adult	1	0	0	0	-
M. alcathoe	M adult	1	0	0	0	-
	F adult	1	0	0	0	-
M. bechsteinii	M Adult	45	0	0	0	-
	M YOY	21	0	0	0	-
	F Adult	2	0	0	0	-
	F YOY	7	0	0	0	-
M. brandtii	M Adult	6	1	2	0.17	2
	M YOY	7	2	4	0.29	2
	F Adult	5	3	4	0.60	1.33
M. dasycneme	M Adult	7	0	0	0	-
	M YOY	1	0	0	0	-
	F Adult	1	0	0	0	-
M. mystacinus	M Adult	9	2	5	0.22	2.5
	M YOY	12	1	2	0.08	2
	F Adult	7	2	4	0.29	2
	F YOY	5	2	7	0.40	3.5
P. auritus	M Adult	12	1	4	0.08	4
	M YOY	3	2	3	0.67	1.5
	F Adult	2	0	0	0	-
	F YOY	2	2	5	1.00	2.5
P. pipistrellus	M Adult	6	0	0	0	-
	M YOY	1	0	0	0	-
	F Adult	1	0	0	0	-

Table ESM2: Since mixed models of intensity were overdispersed when run using a poisson fit, these models were reevaluated using a zero-truncated fit. In all tests, estimates and significance did not differ from the initial model.

VGLM	**Intensity (Overall)**			**Intensity (Temporal)**		
**Species**	**Fixed effect**	**Estimate**	**P**	**Fixed effect**	**Estimate**	**P**
M. daubentonii	Intercept (Ad F)	0.6	<0.01	Intercept (Ad F * day)	-0.02	0.02
	Ad M	0.24	0.12	Ad M * day	0.01	0.42
	YOY F	-0.29	0.24	YOY F * day	-0.03	0.14
	YOY M	0.16	0.40	YOY M * day	0.00	0.91
M. myotis	Intercept (Ad F)	2.00	<0.01	Intercept (Ad F * day)	-0.02	<0.01
	Ad M	-1.14	<0.01	Ad M * day	0.02	0.05
	YOY F	-0.20	<0.01	YOY F * day	-0.01	0.12
	YOY M	-0.67	<0.01	YOY M * day	0.00	0.32
M. nattereri	Intercept (Ad F)	0.26	0.12	Intercept (Ad F * day)	-0.02	0.07
	Ad M	-0.39	0.32	Ad M * day	0.00	0.96
	YOY F	0.40	0.08	YOY F * day	-0.01	0.51
	YOY M	-0.12	0.60	YOY M * day	0.02	0.42

Table ESM3: Location and sampling year were included in all models as random effects. Although no consistent patterns was evident, both location and sampling year did contribute significantly to individual models as summarized in the table below. In *M. myotis*, mite intensity differed across sampling locations when the very small sample set from Brunnen Meyer (n = 4) was included, but did not differ when these were not considered.

	**Species**	**Random effect**	**Prevalence**	**Intensity**
overall per host class	*M. daubentonii*	*location*		
		year	*	
	M. myotis	*location*		*
		loc without BM		
		year		*
	M. nattereri	*location*		
		year		
overall temporal	*M. daubentonii*	*location*		
		year		
	M. myotis	*location*		
		loc without BM		
		year		*
	M. nattereri	*location*	*	
		year		
host class x temporal	*M. daubentonii*	*location*		
		year		
	M. myotis	*location*		
		loc without BM		
		year		
	M. nattereri	*location*	*	
		year		
