# Supplementary material for: Host social organization and mating system shape parasite transmission opportunities in three European bat species
Source: Parasitol Res. 2016 Nov 18;116(2):589–99. doi: 10.1007/s00436-016-5323-8 (PMC5258804; doi:10.1007/s00436-016-5323-8)
Supplement: Supplementary file 2 — Raw data supporting the findings presented in this manuscript. (PDF 1045 kb) [file 436_2016_5323_MOESM2_ESM.pdf]

Raw data to:

van Schaik J[1§] & Kerth G[2] (2016) Host social organization and mating system shape parasite trans
[1] Department of Behavioural Ecology and Evolutionary Genetics, Max Planck Institute for Ornith
[2] Zoological Institute & Museum, Greifswald University, J.-S.-Bach-Str. 11/12, D-17489 Greifswal
§ Corresponding author: Jaap van Schaik; jaapvanschaik@gmail.com; +49 8157 932 416

Day (1 = Aug	Year	Location	Species	Sex	Age	Nmites
4	2012	Esperhohle	Mdaubenton	m	ad	0
4	2012	Esperhohle	Mdaubenton	m	ad	0
4	2012	Esperhohle	Mdaubenton	m	ad	0
4	2012	Esperhohle	Mdaubenton	m	ad	1
4	2012	Esperhohle	Mdaubenton	m	ad	1
4	2012	Esperhohle	Mdaubenton	m	ad	2
4	2012	Esperhohle	Mdaubenton	m	ad	2
4	2012	Esperhohle	Mdaubenton	m	ad	7
4	2012	Esperhohle	Mdaubenton	m	ad	7
4	2012	Esperhohle	Mdaubenton	m	juv	5
4	2012	Esperhohle	Mmyotis	f	ad	3
4	2012	Esperhohle	Mmyotis	f	ad	3
4	2012	Esperhohle	Mmyotis	f	ad	4
4	2012	Esperhohle	Mmyotis	f	ad	5
4	2012	Esperhohle	Mmyotis	f	ad	6
4	2012	Esperhohle	Mmyotis	f	ad	6
4	2012	Esperhohle	Mmyotis	f	ad	6
4	2012	Esperhohle	Mmyotis	f	ad	6
4	2012	Esperhohle	Mmyotis	f	ad	6
4	2012	Esperhohle	Mmyotis	f	ad	6
4	2012	Esperhohle	Mmyotis	f	ad	7
4	2012	Esperhohle	Mmyotis	f	ad	8
4	2012	Esperhohle	Mmyotis	f	ad	9
4	2012	Esperhohle	Mmyotis	f	ad	9
4	2012	Esperhohle	Mmyotis	f	ad	9
4	2012	Esperhohle	Mmyotis	f	ad	10
4	2012	Esperhohle	Mmyotis	f	ad	10
4	2012	Esperhohle	Mmyotis	f	ad	11
4	2012	Esperhohle	Mmyotis	f	ad	11
4	2012	Esperhohle	Mmyotis	f	ad	12
4	2012	Esperhohle	Mmyotis	f	ad	13
4	2012	Esperhohle	Mmyotis	f	ad	14
4	2012	Esperhohle	Mmyotis	f	ad	14
4	2012	Esperhohle	Mmyotis	f	ad	14

4	2012	Esperhohle	Mmyotis	f	ad	15
4	2012	Esperhohle	Mmyotis	f	ad	15
4	2012	Esperhohle	Mmyotis	f	ad	16
4	2012	Esperhohle	Mmyotis	f	ad	18
4	2012	Esperhohle	Mmyotis	f	ad	20
4	2012	Esperhohle	Mmyotis	f	ad	23
4	2012	Esperhohle	Mmyotis	m	ad	0
4	2012	Esperhohle	Mmyotis	m	ad	0
4	2012	Esperhohle	Mmyotis	m	ad	0
4	2012	Esperhohle	Mmyotis	m	ad	0
4	2012	Esperhohle	Mmyotis	m	ad	0
4	2012	Esperhohle	Mmyotis	m	ad	0
4	2012	Esperhohle	Mmyotis	m	ad	0
4	2012	Esperhohle	Mmyotis	m	ad	0
4	2012	Esperhohle	Mmyotis	m	ad	0
4	2012	Esperhohle	Mmyotis	m	ad	0
4	2012	Esperhohle	Mmyotis	m	ad	0
4	2012	Esperhohle	Mmyotis	m	ad	0
4	2012	Esperhohle	Mmyotis	m	ad	0
4	2012	Esperhohle	Mmyotis	m	ad	0
4	2012	Esperhohle	Mmyotis	m	ad	0
4	2012	Esperhohle	Mmyotis	m	ad	0
4	2012	Esperhohle	Mmyotis	m	ad	0
4	2012	Esperhohle	Mmyotis	m	ad	0
4	2012	Esperhohle	Mmyotis	m	ad	0
4	2012	Esperhohle	Mmyotis	m	ad	0
4	2012	Esperhohle	Mmyotis	m	ad	0
4	2012	Esperhohle	Mmyotis	m	ad	0
4	2012	Esperhohle	Mmyotis	m	ad	4
4	2012	Esperhohle	Mmyotis	f	juv	2
4	2012	Esperhohle	Mmyotis	f	juv	6
4	2012	Esperhohle	Mmyotis	f	juv	7
4	2012	Esperhohle	Mmyotis	f	juv	8
4	2012	Esperhohle	Mmyotis	f	juv	14
4	2012	Esperhohle	Mmyotis	f	juv	24
4	2012	Esperhohle	Mmyotis	f	juv	26
4	2012	Esperhohle	Mmyotis	m	juv	0
4	2012	Esperhohle	Mmyotis	m	juv	0
4	2012	Esperhohle	Mmyotis	m	juv	0
4	2012	Esperhohle	Mnattereri	f	ad	5
4	2012	Esperhohle	Mnattereri	m	ad	0

5	2012	Schonstenhol	Mdaubenton	f	ad	3
5	2012	Schonstenhol	Mdaubenton	f	ad	7
5	2012	Schonstenhol	Mdaubenton	m	ad	0
5	2012	Schonstenhol	Mdaubenton	m	ad	0
5	2012	Schonstenhol	Mdaubenton	m	ad	1
5	2012	Schonstenhol	Mdaubenton	m	ad	2
5	2012	Schonstenhol	Mdaubenton	m	ad	2
5	2012	Schonstenhol	Mdaubenton	m	ad	3
5	2012	Schonstenhol	Mdaubenton	m	ad	4
5	2012	Schonstenhol	Mdaubenton	m	ad	9
5	2012	Schonstenhol	Mdaubenton	f	juv	4
5	2012	Schonstenhol	Mmyotis	f	ad	4
5	2012	Schonstenhol	Mmyotis	f	ad	6
5	2012	Schonstenhol	Mmyotis	f	ad	12
5	2012	Schonstenhol	Mmyotis	f	ad	18
5	2012	Schonstenhol	Mmyotis	f	ad	23
5	2012	Schonstenhol	Mmyotis	m	ad	0
5	2012	Schonstenhol	Mmyotis	m	ad	0
5	2012	Schonstenhol	Mmyotis	m	ad	0
5	2012	Schonstenhol	Mmyotis	f	juv	5
5	2012	Schonstenhol	Mmyotis	f	juv	15
5	2012	Schonstenhol	Mmyotis	f	juv	17
5	2012	Schonstenhol	Mmyotis	m	juv	0
5	2012	Schonstenhol	Mmyotis	m	juv	4
5	2012	Schonstenhol	Mmyotis	m	juv	5
5	2012	Schonstenhol	Mmyotis	m	juv	8
5	2012	Schonstenhol	Mnattereri	m	ad	0
5	2012	Schonstenhol	Mnattereri	m	juv	0
17	2012	Esperhohle	Mdaubenton	m	ad	0
17	2012	Esperhohle	Mdaubenton	m	ad	0
17	2012	Esperhohle	Mdaubenton	m	ad	0
17	2012	Esperhohle	Mdaubenton	m	ad	1
17	2012	Esperhohle	Mdaubenton	m	ad	2
17	2012	Esperhohle	Mdaubenton	m	ad	3
17	2012	Esperhohle	Mdaubenton	m	ad	4
17	2012	Esperhohle	Mdaubenton	m	ad	4
17	2012	Esperhohle	Mdaubenton	m	ad	5
17	2012	Esperhohle	Mmyotis	f	ad	1
17	2012	Esperhohle	Mmyotis	f	ad	3
17	2012	Esperhohle	Mmyotis	f	ad	4
17	2012	Esperhohle	Mmyotis	f	ad	7

17	2012	Esperhohle	Mmyotis	f	ad	8
17	2012	Esperhohle	Mmyotis	f	ad	8
17	2012	Esperhohle	Mmyotis	f	ad	9
17	2012	Esperhohle	Mmyotis	f	ad	10
17	2012	Esperhohle	Mmyotis	f	ad	10
17	2012	Esperhohle	Mmyotis	f	ad	12
17	2012	Esperhohle	Mmyotis	m	ad	0
17	2012	Esperhohle	Mmyotis	m	ad	0
17	2012	Esperhohle	Mmyotis	m	ad	0
17	2012	Esperhohle	Mmyotis	m	ad	0
17	2012	Esperhohle	Mmyotis	m	ad	0
17	2012	Esperhohle	Mmyotis	m	ad	0
17	2012	Esperhohle	Mmyotis	m	ad	0
17	2012	Esperhohle	Mmyotis	m	ad	0
17	2012	Esperhohle	Mmyotis	f	juv	2
17	2012	Esperhohle	Mmyotis	f	juv	9
17	2012	Esperhohle	Mmyotis	f	juv	17
17	2012	Esperhohle	Mmyotis	m	juv	9
17	2012	Esperhohle	Mnattereri	m	ad	0
17	2012	Esperhohle	Mnattereri	m	ad	0
17	2012	Esperhohle	Mnattereri	m	ad	0
17	2012	Esperhohle	Mnattereri	m	ad	0
17	2012	Esperhohle	Mnattereri	m	ad	0
17	2012	Esperhohle	Mnattereri	m	ad	0
17	2012	Esperhohle	Mnattereri	m	juv	0
18	2012	Schonstenhol	Mdaubenton	f	ad	2
18	2012	Schonstenhol	Mdaubenton	m	ad	0
18	2012	Schonstenhol	Mdaubenton	m	ad	0
18	2012	Schonstenhol	Mdaubenton	m	ad	0
18	2012	Schonstenhol	Mdaubenton	m	ad	0
18	2012	Schonstenhol	Mdaubenton	m	ad	1
18	2012	Schonstenhol	Mdaubenton	m	ad	1
18	2012	Schonstenhol	Mdaubenton	m	ad	2
18	2012	Schonstenhol	Mdaubenton	m	ad	2
18	2012	Schonstenhol	Mdaubenton	m	ad	3
18	2012	Schonstenhol	Mdaubenton	m	ad	5
18	2012	Schonstenhol	Mdaubenton	m	ad	7
18	2012	Schonstenhol	Mdaubenton	f	juv	4
18	2012	Schonstenhol	Mdaubenton	m	juv	0
18	2012	Schonstenhol	Mmyotis	f	ad	3
18	2012	Schonstenhol	Mmyotis	f	ad	5

18	2012	Schonstenhol	Mmyotis	f	ad	10
18	2012	Schonstenhol	Mmyotis	f	ad	12
18	2012	Schonstenhol	Mmyotis	f	ad	16
18	2012	Schonstenhol	Mmyotis	m	ad	0
18	2012	Schonstenhol	Mmyotis	f	juv	2
18	2012	Schonstenhol	Mmyotis	f	juv	4
18	2012	Schonstenhol	Mmyotis	f	juv	15
18	2012	Schonstenhol	Mmyotis	m	juv	0
18	2012	Schonstenhol	Mmyotis	m	juv	4
18	2012	Schonstenhol	Mmyotis	m	juv	6
18	2012	Schonstenhol	Mmyotis	m	juv	9
18	2012	Schonstenhol	Mnattereri	f	ad	0
18	2012	Schonstenhol	Mnattereri	f	ad	0
18	2012	Schonstenhol	Mnattereri	m	ad	0
18	2012	Schonstenhol	Mnattereri	m	ad	0
18	2012	Schonstenhol	Mnattereri	m	ad	0
18	2012	Schonstenhol	Mnattereri	m	ad	0
18	2012	Schonstenhol	Mnattereri	m	ad	0
18	2012	Schonstenhol	Mnattereri	m	ad	0
18	2012	Schonstenhol	Mnattereri	m	ad	0
18	2012	Schonstenhol	Mnattereri	m	ad	0
18	2012	Schonstenhol	Mnattereri	m	ad	0
18	2012	Schonstenhol	Mnattereri	m	ad	0
18	2012	Schonstenhol	Mnattereri	m	ad	0
18	2012	Schonstenhol	Mnattereri	m	ad	0
18	2012	Schonstenhol	Mnattereri	m	ad	0
18	2012	Schonstenhol	Mnattereri	m	ad	0
18	2012	Schonstenhol	Mnattereri	m	ad	0
18	2012	Schonstenhol	Mnattereri	m	ad	0
18	2012	Schonstenhol	Mnattereri	m	ad	0
18	2012	Schonstenhol	Mnattereri	m	ad	0
18	2012	Schonstenhol	Mnattereri	m	ad	0
18	2012	Schonstenhol	Mnattereri	m	ad	0
18	2012	Schonstenhol	Mnattereri	m	ad	0
18	2012	Schonstenhol	Mnattereri	m	ad	0
18	2012	Schonstenhol	Mnattereri	m	ad	0
18	2012	Schonstenhol	Mnattereri	m	ad	0
18	2012	Schonstenhol	Mnattereri	m	ad	0
18	2012	Schonstenhol	Mnattereri	m	ad	0
18	2012	Schonstenhol	Mnattereri	f	juv	3
18	2012	Schonstenhol	Mnattereri	m	juv	0

18	2012	Schonstenhol	Mnattereri	m	juv	0
18	2012	Schonstenhol	Mnattereri	m	juv	0
18	2012	Schonstenhol	Mnattereri	m	juv	0
18	2012	Schonstenhol	Mnattereri	m	juv	0
18	2012	Schonstenhol	Mnattereri	m	juv	1
18	2012	Schonstenhol	Mnattereri	m	juv	2
23	2012	Brunnen Mey	Mdaubenton	f	ad	0
23	2012	Brunnen Mey	Mdaubenton	f	ad	0
23	2012	Brunnen Mey	Mdaubenton	f	ad	0
23	2012	Brunnen Mey	Mdaubenton	f	ad	1
23	2012	Brunnen Mey	Mdaubenton	f	ad	1
23	2012	Brunnen Mey	Mdaubenton	f	ad	1
23	2012	Brunnen Mey	Mdaubenton	f	ad	2
23	2012	Brunnen Mey	Mdaubenton	m	ad	0
23	2012	Brunnen Mey	Mdaubenton	m	ad	0
23	2012	Brunnen Mey	Mdaubenton	m	ad	0
23	2012	Brunnen Mey	Mdaubenton	m	ad	0
23	2012	Brunnen Mey	Mdaubenton	m	ad	1
23	2012	Brunnen Mey	Mdaubenton	m	ad	1
23	2012	Brunnen Mey	Mdaubenton	m	ad	1
23	2012	Brunnen Mey	Mdaubenton	m	ad	1
23	2012	Brunnen Mey	Mdaubenton	m	ad	1
23	2012	Brunnen Mey	Mdaubenton	m	ad	1
23	2012	Brunnen Mey	Mdaubenton	m	ad	2
23	2012	Brunnen Mey	Mdaubenton	m	ad	3
23	2012	Brunnen Mey	Mdaubenton	m	ad	3
23	2012	Brunnen Mey	Mdaubenton	m	ad	4
23	2012	Brunnen Mey	Mdaubenton	m	ad	4
23	2012	Brunnen Mey	Mdaubenton	m	ad	5
23	2012	Brunnen Mey	Mdaubenton	f	juv	1
23	2012	Brunnen Mey	Mdaubenton	f	juv	2
23	2012	Brunnen Mey	Mdaubenton	f	juv	3
23	2012	Brunnen Mey	Mdaubenton	f	juv	3
23	2012	Brunnen Mey	Mdaubenton	m	juv	0
23	2012	Brunnen Mey	Mdaubenton	m	juv	0
23	2012	Brunnen Mey	Mdaubenton	m	juv	0
23	2012	Brunnen Mey	Mdaubenton	m	juv	1
23	2012	Brunnen Mey	Mdaubenton	m	juv	1
23	2012	Brunnen Mey	Mdaubenton	m	juv	2
23	2012	Brunnen Mey	Mdaubenton	m	juv	2
23	2012	Brunnen Mey	Mdaubenton	m	juv	2

23	2012	Brunnen Mey	Mdaubenton	m	juv	3
23	2012	Brunnen Mey	Mdaubenton	m	juv	3
23	2012	Brunnen Mey	Mdaubenton	m	juv	4
23	2012	Brunnen Mey	Mdaubenton	m	juv	5
23	2012	Brunnen Mey	Mnattereri	f	ad	0
23	2012	Brunnen Mey	Mnattereri	f	ad	0
23	2012	Brunnen Mey	Mnattereri	m	ad	0
23	2012	Brunnen Mey	Mnattereri	f	juv	5
23	2012	Brunnen Mey	Mnattereri	m	juv	0
23	2012	Brunnen Mey	Mnattereri	m	juv	0
23	2012	Brunnen Mey	Mnattereri	m	juv	1
26	2011	Esperhohle	Mdaubenton	m	ad	0
26	2011	Esperhohle	Mdaubenton	m	ad	1
26	2011	Esperhohle	Mdaubenton	m	ad	1
26	2011	Esperhohle	Mdaubenton	m	juv	0
26	2011	Esperhohle	Mmyotis	f	ad	5
26	2011	Esperhohle	Mmyotis	f	ad	6
26	2011	Esperhohle	Mmyotis	f	ad	8
26	2011	Esperhohle	Mmyotis	f	ad	8
26	2011	Esperhohle	Mmyotis	f	ad	11
26	2011	Esperhohle	Mmyotis	f	ad	16
26	2011	Esperhohle	Mmyotis	m	ad	0
26	2011	Esperhohle	Mmyotis	m	ad	0
26	2011	Esperhohle	Mmyotis	m	ad	0
26	2011	Esperhohle	Mmyotis	m	ad	0
26	2011	Esperhohle	Mmyotis	m	ad	0
26	2011	Esperhohle	Mmyotis	m	ad	0
26	2011	Esperhohle	Mmyotis	m	ad	0
26	2011	Esperhohle	Mmyotis	m	ad	0
26	2011	Esperhohle	Mmyotis	m	ad	0
26	2011	Esperhohle	Mmyotis	m	ad	0
26	2011	Esperhohle	Mmyotis	m	ad	0
26	2011	Esperhohle	Mmyotis	m	ad	0
26	2011	Esperhohle	Mmyotis	m	ad	0
26	2011	Esperhohle	Mmyotis	m	ad	0
26	2011	Esperhohle	Mmyotis	m	ad	1
26	2011	Esperhohle	Mmyotis	m	ad	2
26	2011	Esperhohle	Mmyotis	m	ad	2
26	2011	Esperhohle	Mmyotis	m	ad	2
26	2011	Esperhohle	Mmyotis	m	ad	3
26	2011	Esperhohle	Mmyotis	f	juv	0

26	2011	Esperhohle	Mmyotis	f	juv	0
26	2011	Esperhohle	Mmyotis	f	juv	2
26	2011	Esperhohle	Mmyotis	f	juv	4
26	2011	Esperhohle	Mmyotis	f	juv	5
26	2011	Esperhohle	Mmyotis	f	juv	8
26	2011	Esperhohle	Mmyotis	f	juv	8
26	2011	Esperhohle	Mmyotis	f	juv	13
26	2011	Esperhohle	Mmyotis	m	juv	1
26	2011	Esperhohle	Mmyotis	m	juv	1
26	2011	Esperhohle	Mmyotis	m	juv	2
26	2011	Esperhohle	Mmyotis	m	juv	3
26	2011	Esperhohle	Mmyotis	m	juv	4
26	2011	Esperhohle	Mmyotis	m	juv	4
26	2011	Esperhohle	Mmyotis	m	juv	6
26	2011	Esperhohle	Mnattereri	f	ad	0
26	2011	Esperhohle	Mnattereri	m	ad	0
27	2011	Schonstenhol	Mdaubenton	m	ad	0
27	2011	Schonstenhol	Mdaubenton	m	ad	1
27	2011	Schonstenhol	Mdaubenton	m	ad	2
27	2011	Schonstenhol	Mdaubenton	m	juv	0
27	2011	Schonstenhol	Mdaubenton	m	juv	1
27	2011	Schonstenhol	Mmyotis	f	ad	0
27	2011	Schonstenhol	Mmyotis	f	ad	0
27	2011	Schonstenhol	Mmyotis	f	ad	0
27	2011	Schonstenhol	Mmyotis	f	ad	2
27	2011	Schonstenhol	Mmyotis	f	ad	2
27	2011	Schonstenhol	Mmyotis	m	ad	0
27	2011	Schonstenhol	Mmyotis	m	ad	0
27	2011	Schonstenhol	Mmyotis	m	ad	2
27	2011	Schonstenhol	Mmyotis	f	juv	1
27	2011	Schonstenhol	Mmyotis	f	juv	2
27	2011	Schonstenhol	Mmyotis	m	juv	0
27	2011	Schonstenhol	Mmyotis	m	juv	0
27	2011	Schonstenhol	Mmyotis	m	juv	2
27	2011	Schonstenhol	Mmyotis	m	juv	3
27	2011	Schonstenhol	Mmyotis	m	juv	6
27	2011	Schonstenhol	Mnattereri	m	ad	0
27	2011	Schonstenhol	Mnattereri	m	ad	0
27	2011	Schonstenhol	Mnattereri	m	ad	0
27	2011	Schonstenhol	Mnattereri	m	ad	0
27	2011	Schonstenhol	Mnattereri	m	ad	0

27	2011	Schonstenhol	Mnattereri	m	ad	0
27	2011	Schonstenhol	Mnattereri	m	ad	0
27	2011	Schonstenhol	Mnattereri	m	ad	0
27	2011	Schonstenhol	Mnattereri	m	ad	0
27	2011	Schonstenhol	Mnattereri	m	ad	0
27	2011	Schonstenhol	Mnattereri	m	ad	0
27	2011	Schonstenhol	Mnattereri	m	ad	0
27	2011	Schonstenhol	Mnattereri	m	ad	0
27	2011	Schonstenhol	Mnattereri	m	ad	0
27	2011	Schonstenhol	Mnattereri	m	ad	0
27	2011	Schonstenhol	Mnattereri	m	ad	0
27	2011	Schonstenhol	Mnattereri	m	ad	0
27	2011	Schonstenhol	Mnattereri	m	ad	0
27	2011	Schonstenhol	Mnattereri	m	ad	0
27	2011	Schonstenhol	Mnattereri	m	ad	0
27	2011	Schonstenhol	Mnattereri	f	juv	1
27	2011	Schonstenhol	Mnattereri	m	juv	0
27	2011	Schonstenhol	Mnattereri	m	juv	0
27	2011	Schonstenhol	Mnattereri	m	juv	0
29	2011	Brunnen Mey	Mdaubenton	f	ad	0
29	2011	Brunnen Mey	Mdaubenton	f	ad	0
29	2011	Brunnen Mey	Mdaubenton	f	ad	0
29	2011	Brunnen Mey	Mdaubenton	f	ad	0
29	2011	Brunnen Mey	Mdaubenton	f	ad	0
29	2011	Brunnen Mey	Mdaubenton	f	ad	1
29	2011	Brunnen Mey	Mdaubenton	f	ad	1
29	2011	Brunnen Mey	Mdaubenton	f	ad	2
29	2011	Brunnen Mey	Mdaubenton	f	ad	3
29	2011	Brunnen Mey	Mdaubenton	f	ad	4
29	2011	Brunnen Mey	Mdaubenton	f	ad	5
29	2011	Brunnen Mey	Mdaubenton	m	ad	0
29	2011	Brunnen Mey	Mdaubenton	m	ad	0
29	2011	Brunnen Mey	Mdaubenton	m	ad	0
29	2011	Brunnen Mey	Mdaubenton	m	ad	0
29	2011	Brunnen Mey	Mdaubenton	m	ad	0
29	2011	Brunnen Mey	Mdaubenton	m	ad	0
29	2011	Brunnen Mey	Mdaubenton	m	ad	0
29	2011	Brunnen Mey	Mdaubenton	m	ad	0
29	2011	Brunnen Mey	Mdaubenton	m	ad	0
29	2011	Brunnen Mey	Mdaubenton	m	ad	0
29	2011	Brunnen Mey	Mdaubenton	m	ad	0

29	2011	Brunnen Mey	Mdaubenton	m	ad	0
29	2011	Brunnen Mey	Mdaubenton	m	ad	0
29	2011	Brunnen Mey	Mdaubenton	m	ad	0
29	2011	Brunnen Mey	Mdaubenton	m	ad	1
29	2011	Brunnen Mey	Mdaubenton	m	ad	1
29	2011	Brunnen Mey	Mdaubenton	m	ad	1
29	2011	Brunnen Mey	Mdaubenton	m	ad	1
29	2011	Brunnen Mey	Mdaubenton	m	ad	1
29	2011	Brunnen Mey	Mdaubenton	m	ad	2
29	2011	Brunnen Mey	Mdaubenton	m	ad	2
29	2011	Brunnen Mey	Mdaubenton	m	ad	2
29	2011	Brunnen Mey	Mdaubenton	m	ad	2
29	2011	Brunnen Mey	Mdaubenton	m	ad	3
29	2011	Brunnen Mey	Mdaubenton	m	ad	3
29	2011	Brunnen Mey	Mdaubenton	m	ad	3
29	2011	Brunnen Mey	Mdaubenton	m	ad	3
29	2011	Brunnen Mey	Mdaubenton	m	ad	5
29	2011	Brunnen Mey	Mdaubenton	m	ad	5
29	2011	Brunnen Mey	Mdaubenton	f	juv	1
29	2011	Brunnen Mey	Mdaubenton	f	juv	2
29	2011	Brunnen Mey	Mdaubenton	m	juv	0
29	2011	Brunnen Mey	Mdaubenton	m	juv	0
29	2011	Brunnen Mey	Mdaubenton	m	juv	0
29	2011	Brunnen Mey	Mdaubenton	m	juv	1
29	2011	Brunnen Mey	Mdaubenton	m	juv	4
29	2011	Brunnen Mey	Mdaubenton	m	juv	4
29	2011	Brunnen Mey	Mmyotis	f	ad	0
29	2011	Brunnen Mey	Mnattereri	f	ad	0
29	2011	Brunnen Mey	Mnattereri	f	ad	0
29	2011	Brunnen Mey	Mnattereri	f	ad	0
29	2011	Brunnen Mey	Mnattereri	f	ad	1
29	2011	Brunnen Mey	Mnattereri	f	ad	1
29	2011	Brunnen Mey	Mnattereri	f	ad	3
29	2011	Brunnen Mey	Mnattereri	m	ad	0
29	2011	Brunnen Mey	Mnattereri	m	ad	0
29	2011	Brunnen Mey	Mnattereri	m	ad	0
29	2011	Brunnen Mey	Mnattereri	m	ad	0
29	2011	Brunnen Mey	Mnattereri	m	ad	0
29	2011	Brunnen Mey	Mnattereri	m	ad	0
29	2011	Brunnen Mey	Mnattereri	f	juv	0
29	2011	Brunnen Mey	Mnattereri	f	juv	0

29	2011	Brunnen Mey	Mnattereri	f	juv	6
29	2011	Brunnen Mey	Mnattereri	m	juv	0
29	2011	Brunnen Mey	Mnattereri	m	juv	0
29	2011	Brunnen Mey	Mnattereri	m	juv	0
29	2011	Brunnen Mey	Mnattereri	m	juv	1
29	2011	Brunnen Mey	Mnattereri	m	juv	1
29	2011	Brunnen Mey	Mnattereri	m	juv	1
29	2011	Brunnen Mey	Mnattereri	m	juv	1
29	2011	Brunnen Mey	Mnattereri	m	juv	1
29	2011	Brunnen Mey	Mnattereri	m	juv	1
29	2011	Brunnen Mey	Mnattereri	m	juv	3
32	2012	Schonstenhol	Mdaubenton	m	ad	0
32	2012	Schonstenhol	Mdaubenton	m	ad	0
32	2012	Schonstenhol	Mdaubenton	m	ad	0
32	2012	Schonstenhol	Mdaubenton	m	ad	2
32	2012	Schonstenhol	Mdaubenton	m	ad	2
32	2012	Schonstenhol	Mdaubenton	m	ad	2
32	2012	Schonstenhol	Mdaubenton	f	juv	0
32	2012	Schonstenhol	Mdaubenton	f	juv	1
32	2012	Schonstenhol	Mdaubenton	f	juv	2
32	2012	Schonstenhol	Mdaubenton	m	juv	1
32	2012	Schonstenhol	Mdaubenton	m	juv	2
32	2012	Schonstenhol	Mmyotis	f	ad	9
32	2012	Schonstenhol	Mmyotis	f	ad	10
32	2012	Schonstenhol	Mnattereri	f	ad	0
32	2012	Schonstenhol	Mnattereri	m	ad	0
32	2012	Schonstenhol	Mnattereri	m	ad	0
32	2012	Schonstenhol	Mnattereri	m	ad	1
32	2012	Schonstenhol	Mnattereri	m	juv	0
33	2012	Esperhohle	Mdaubenton	f	ad	0
33	2012	Esperhohle	Mdaubenton	f	ad	3
33	2012	Esperhohle	Mdaubenton	m	ad	0
33	2012	Esperhohle	Mdaubenton	m	ad	0
33	2012	Esperhohle	Mdaubenton	m	ad	0
33	2012	Esperhohle	Mdaubenton	m	ad	0
33	2012	Esperhohle	Mdaubenton	m	ad	1
33	2012	Esperhohle	Mdaubenton	m	ad	1
33	2012	Esperhohle	Mdaubenton	f	juv	0
33	2012	Esperhohle	Mdaubenton	f	juv	2
33	2012	Esperhohle	Mdaubenton	f	juv	2
33	2012	Esperhohle	Mdaubenton	m	juv	0

33	2012	Esperhohle	Mdaubenton	m	juv	0
33	2012	Esperhohle	Mdaubenton	m	juv	2
33	2012	Esperhohle	Mdaubenton	m	juv	5
33	2012	Esperhohle	Mmyotis	f	ad	1
33	2012	Esperhohle	Mmyotis	f	ad	2
33	2012	Esperhohle	Mmyotis	f	ad	2
33	2012	Esperhohle	Mmyotis	f	ad	2
33	2012	Esperhohle	Mmyotis	f	ad	2
33	2012	Esperhohle	Mmyotis	f	ad	3
33	2012	Esperhohle	Mmyotis	f	ad	3
33	2012	Esperhohle	Mmyotis	f	ad	3
33	2012	Esperhohle	Mmyotis	f	ad	4
33	2012	Esperhohle	Mmyotis	f	ad	4
33	2012	Esperhohle	Mmyotis	f	ad	4
33	2012	Esperhohle	Mmyotis	f	ad	5
33	2012	Esperhohle	Mmyotis	f	ad	6
33	2012	Esperhohle	Mmyotis	f	ad	6
33	2012	Esperhohle	Mmyotis	f	ad	7
33	2012	Esperhohle	Mmyotis	f	ad	8
33	2012	Esperhohle	Mmyotis	f	ad	8
33	2012	Esperhohle	Mmyotis	f	ad	8
33	2012	Esperhohle	Mmyotis	f	ad	8
33	2012	Esperhohle	Mmyotis	f	ad	9
33	2012	Esperhohle	Mmyotis	f	ad	9
33	2012	Esperhohle	Mmyotis	f	ad	10
33	2012	Esperhohle	Mmyotis	f	ad	11
33	2012	Esperhohle	Mmyotis	f	ad	12
33	2012	Esperhohle	Mmyotis	f	ad	15
33	2012	Esperhohle	Mmyotis	f	ad	15
33	2012	Esperhohle	Mmyotis	f	ad	23
33	2012	Esperhohle	Mmyotis	f	ad	29
33	2012	Esperhohle	Mmyotis	m	ad	0
33	2012	Esperhohle	Mmyotis	m	ad	0
33	2012	Esperhohle	Mmyotis	m	ad	0
33	2012	Esperhohle	Mmyotis	m	ad	0
33	2012	Esperhohle	Mmyotis	m	ad	0
33	2012	Esperhohle	Mmyotis	m	ad	0
33	2012	Esperhohle	Mmyotis	m	ad	0
33	2012	Esperhohle	Mmyotis	m	ad	0
33	2012	Esperhohle	Mmyotis	m	ad	0
33	2012	Esperhohle	Mmyotis	m	ad	0

33	2012	Esperhohle	Mmyotis	m	ad	0
33	2012	Esperhohle	Mmyotis	m	ad	3
33	2012	Esperhohle	Mmyotis	f	juv	1
33	2012	Esperhohle	Mmyotis	f	juv	1
33	2012	Esperhohle	Mmyotis	f	juv	3
33	2012	Esperhohle	Mmyotis	f	juv	3
33	2012	Esperhohle	Mmyotis	f	juv	3
33	2012	Esperhohle	Mmyotis	f	juv	5
33	2012	Esperhohle	Mmyotis	f	juv	5
33	2012	Esperhohle	Mmyotis	f	juv	6
33	2012	Esperhohle	Mmyotis	f	juv	6
33	2012	Esperhohle	Mmyotis	f	juv	8
33	2012	Esperhohle	Mmyotis	f	juv	10
33	2012	Esperhohle	Mmyotis	f	juv	13
33	2012	Esperhohle	Mmyotis	m	juv	0
33	2012	Esperhohle	Mmyotis	m	juv	0
33	2012	Esperhohle	Mmyotis	m	juv	0
33	2012	Esperhohle	Mmyotis	m	juv	0
33	2012	Esperhohle	Mmyotis	m	juv	0
33	2012	Esperhohle	Mmyotis	m	juv	2
33	2012	Esperhohle	Mmyotis	m	juv	3
33	2012	Esperhohle	Mmyotis	m	juv	5
33	2012	Esperhohle	Mmyotis	m	juv	6
33	2012	Esperhohle	Mmyotis	m	juv	7
33	2012	Esperhohle	Mnattereri	f	ad	0
33	2012	Esperhohle	Mnattereri	m	ad	0
33	2012	Esperhohle	Mnattereri	m	ad	0
33	2012	Esperhohle	Mnattereri	m	ad	0
33	2012	Esperhohle	Mnattereri	m	juv	0
36	2012	Brunnen Mey	Mdaubenton	f	ad	0
36	2012	Brunnen Mey	Mdaubenton	f	ad	0
36	2012	Brunnen Mey	Mdaubenton	f	ad	0
36	2012	Brunnen Mey	Mdaubenton	f	ad	0
36	2012	Brunnen Mey	Mdaubenton	f	ad	0
36	2012	Brunnen Mey	Mdaubenton	f	ad	0
36	2012	Brunnen Mey	Mdaubenton	f	ad	1
36	2012	Brunnen Mey	Mdaubenton	f	ad	1
36	2012	Brunnen Mey	Mdaubenton	f	ad	1
36	2012	Brunnen Mey	Mdaubenton	f	ad	1
36	2012	Brunnen Mey	Mdaubenton	f	ad	2
36	2012	Brunnen Mey	Mdaubenton	f	ad	2

36	2012	Brunnen Mey	Mdaubenton	f	ad	3
36	2012	Brunnen Mey	Mdaubenton	f	ad	4
36	2012	Brunnen Mey	Mdaubenton	f	ad	6
36	2012	Brunnen Mey	Mdaubenton	m	ad	0
36	2012	Brunnen Mey	Mdaubenton	m	ad	0
36	2012	Brunnen Mey	Mdaubenton	m	ad	0
36	2012	Brunnen Mey	Mdaubenton	m	ad	0
36	2012	Brunnen Mey	Mdaubenton	m	ad	0
36	2012	Brunnen Mey	Mdaubenton	m	ad	0
36	2012	Brunnen Mey	Mdaubenton	m	ad	0
36	2012	Brunnen Mey	Mdaubenton	m	ad	0
36	2012	Brunnen Mey	Mdaubenton	m	ad	0
36	2012	Brunnen Mey	Mdaubenton	m	ad	1
36	2012	Brunnen Mey	Mdaubenton	m	ad	2
36	2012	Brunnen Mey	Mdaubenton	m	ad	2
36	2012	Brunnen Mey	Mdaubenton	m	ad	2
36	2012	Brunnen Mey	Mdaubenton	m	ad	5
36	2012	Brunnen Mey	Mdaubenton	f	juv	1
36	2012	Brunnen Mey	Mdaubenton	f	juv	1
36	2012	Brunnen Mey	Mdaubenton	f	juv	2
36	2012	Brunnen Mey	Mdaubenton	f	juv	2
36	2012	Brunnen Mey	Mdaubenton	m	juv	0
36	2012	Brunnen Mey	Mdaubenton	m	juv	0
36	2012	Brunnen Mey	Mdaubenton	m	juv	0
36	2012	Brunnen Mey	Mdaubenton	m	juv	0
36	2012	Brunnen Mey	Mdaubenton	m	juv	1
36	2012	Brunnen Mey	Mdaubenton	m	juv	1
36	2012	Brunnen Mey	Mdaubenton	m	juv	2
36	2012	Brunnen Mey	Mdaubenton	m	juv	2
36	2012	Brunnen Mey	Mmyotis	f	juv	0
36	2012	Brunnen Mey	Mnattereri	f	ad	0
36	2012	Brunnen Mey	Mnattereri	f	ad	0
36	2012	Brunnen Mey	Mnattereri	f	ad	0
36	2012	Brunnen Mey	Mnattereri	f	ad	0
36	2012	Brunnen Mey	Mnattereri	f	ad	0
36	2012	Brunnen Mey	Mnattereri	f	ad	0
36	2012	Brunnen Mey	Mnattereri	f	ad	1
36	2012	Brunnen Mey	Mnattereri	f	ad	1
36	2012	Brunnen Mey	Mnattereri	m	ad	0
36	2012	Brunnen Mey	Mnattereri	m	ad	0
36	2012	Brunnen Mey	Mnattereri	m	ad	0

36	2012	Brunnen Mey	Mnattereri	m	ad	0
36	2012	Brunnen Mey	Mnattereri	m	ad	3
36	2012	Brunnen Mey	Mnattereri	m	juv	0
36	2012	Brunnen Mey	Mnattereri	m	juv	0
36	2012	Brunnen Mey	Mnattereri	m	juv	0
36	2012	Brunnen Mey	Mnattereri	m	juv	0
36	2012	Brunnen Mey	Mnattereri	m	juv	2
36	2012	Brunnen Mey	Mnattereri	m	juv	4
37	2011	Schonstenhol	Mdaubenton	f	ad	0
37	2011	Schonstenhol	Mdaubenton	m	ad	0
37	2011	Schonstenhol	Mdaubenton	m	ad	0
37	2011	Schonstenhol	Mdaubenton	m	ad	0
37	2011	Schonstenhol	Mmyotis	f	ad	0
37	2011	Schonstenhol	Mmyotis	m	ad	0
37	2011	Schonstenhol	Mmyotis	m	ad	0
37	2011	Schonstenhol	Mmyotis	m	ad	1
37	2011	Schonstenhol	Mmyotis	m	ad	1
37	2011	Schonstenhol	Mmyotis	m	ad	1
37	2011	Schonstenhol	Mmyotis	m	ad	2
37	2011	Schonstenhol	Mmyotis	m	ad	3
37	2011	Schonstenhol	Mmyotis	f	juv	3
37	2011	Schonstenhol	Mmyotis	f	juv	5
37	2011	Schonstenhol	Mmyotis	m	juv	3
37	2011	Schonstenhol	Mnattereri	f	ad	0
37	2011	Schonstenhol	Mnattereri	f	ad	0
37	2011	Schonstenhol	Mnattereri	f	ad	0
37	2011	Schonstenhol	Mnattereri	f	ad	0
37	2011	Schonstenhol	Mnattereri	f	ad	0
37	2011	Schonstenhol	Mnattereri	f	ad	0
37	2011	Schonstenhol	Mnattereri	f	ad	0
37	2011	Schonstenhol	Mnattereri	f	ad	0
37	2011	Schonstenhol	Mnattereri	f	ad	1
37	2011	Schonstenhol	Mnattereri	f	ad	1
37	2011	Schonstenhol	Mnattereri	f	ad	2
37	2011	Schonstenhol	Mnattereri	f	ad	4
37	2011	Schonstenhol	Mnattereri	m	ad	0
37	2011	Schonstenhol	Mnattereri	m	ad	0
37	2011	Schonstenhol	Mnattereri	m	ad	0
37	2011	Schonstenhol	Mnattereri	m	ad	0
37	2011	Schonstenhol	Mnattereri	m	ad	0
37	2011	Schonstenhol	Mnattereri	m	ad	0

37	2011	Schonstenhol	Mnattereri	m	ad	0
37	2011	Schonstenhol	Mnattereri	m	ad	0
37	2011	Schonstenhol	Mnattereri	m	ad	0
37	2011	Schonstenhol	Mnattereri	m	ad	0
37	2011	Schonstenhol	Mnattereri	m	ad	0
37	2011	Schonstenhol	Mnattereri	m	ad	0
37	2011	Schonstenhol	Mnattereri	m	ad	0
37	2011	Schonstenhol	Mnattereri	m	ad	0
37	2011	Schonstenhol	Mnattereri	m	ad	0
37	2011	Schonstenhol	Mnattereri	m	ad	0
37	2011	Schonstenhol	Mnattereri	m	ad	0
37	2011	Schonstenhol	Mnattereri	m	ad	0
37	2011	Schonstenhol	Mnattereri	m	ad	0
37	2011	Schonstenhol	Mnattereri	m	ad	0
37	2011	Schonstenhol	Mnattereri	m	ad	0
37	2011	Schonstenhol	Mnattereri	m	ad	0
37	2011	Schonstenhol	Mnattereri	m	ad	0
37	2011	Schonstenhol	Mnattereri	m	ad	0
37	2011	Schonstenhol	Mnattereri	m	ad	0
37	2011	Schonstenhol	Mnattereri	m	ad	0
37	2011	Schonstenhol	Mnattereri	m	ad	0
37	2011	Schonstenhol	Mnattereri	m	ad	0
37	2011	Schonstenhol	Mnattereri	m	ad	0
37	2011	Schonstenhol	Mnattereri	m	ad	0
37	2011	Schonstenhol	Mnattereri	m	ad	0
37	2011	Schonstenhol	Mnattereri	m	ad	0
37	2011	Schonstenhol	Mnattereri	m	ad	0
37	2011	Schonstenhol	Mnattereri	m	ad	0
37	2011	Schonstenhol	Mnattereri	m	ad	0
37	2011	Schonstenhol	Mnattereri	m	ad	0
37	2011	Schonstenhol	Mnattereri	m	ad	0
37	2011	Schonstenhol	Mnattereri	m	ad	0
37	2011	Schonstenhol	Mnattereri	m	ad	0
37	2011	Schonstenhol	Mnattereri	m	ad	0
37	2011	Schonstenhol	Mnattereri	m	ad	0
37	2011	Schonstenhol	Mnattereri	m	ad	0
37	2011	Schonstenhol	Mnattereri	m	ad	0
37	2011	Schonstenhol	Mnattereri	m	ad	0
37	2011	Schonstenhol	Mnattereri	m	ad	0
37	2011	Schonstenhol	Mnattereri	m	ad	0
37	2011	Schonstenhol	Mnattereri	m	ad	0

37	2011	Schonstenhol	Mnattereri	m	ad	0
37	2011	Schonstenhol	Mnattereri	m	ad	0
37	2011	Schonstenhol	Mnattereri	m	ad	1
37	2011	Schonstenhol	Mnattereri	m	ad	1
37	2011	Schonstenhol	Mnattereri	m	ad	1
37	2011	Schonstenhol	Mnattereri	m	ad	2
37	2011	Schonstenhol	Mnattereri	f	juv	0
37	2011	Schonstenhol	Mnattereri	f	juv	0
37	2011	Schonstenhol	Mnattereri	f	juv	0
37	2011	Schonstenhol	Mnattereri	f	juv	0
37	2011	Schonstenhol	Mnattereri	f	juv	0
37	2011	Schonstenhol	Mnattereri	f	juv	0
37	2011	Schonstenhol	Mnattereri	f	juv	2
37	2011	Schonstenhol	Mnattereri	m	juv	0
37	2011	Schonstenhol	Mnattereri	m	juv	0
37	2011	Schonstenhol	Mnattereri	m	juv	0
37	2011	Schonstenhol	Mnattereri	m	juv	0
37	2011	Schonstenhol	Mnattereri	m	juv	0
37	2011	Schonstenhol	Mnattereri	m	juv	0
37	2011	Schonstenhol	Mnattereri	m	juv	0
37	2011	Schonstenhol	Mnattereri	m	juv	0
37	2011	Schonstenhol	Mnattereri	m	juv	0
37	2011	Schonstenhol	Mnattereri	m	juv	0
37	2011	Schonstenhol	Mnattereri	m	juv	0
37	2011	Schonstenhol	Mnattereri	m	juv	0
37	2011	Schonstenhol	Mnattereri	m	juv	0
37	2011	Schonstenhol	Mnattereri	m	juv	0
37	2011	Schonstenhol	Mnattereri	m	juv	0
37	2011	Schonstenhol	Mnattereri	m	juv	1
37	2011	Schonstenhol	Mnattereri	m	juv	1
37	2011	Schonstenhol	Mnattereri	m	juv	2
37	2011	Schonstenhol	Mnattereri	m	juv	5
38	2011	Esperhohle	Mdaubenton	f	ad	0
38	2011	Esperhohle	Mmyotis	f	ad	1
38	2011	Esperhohle	Mmyotis	f	ad	2
38	2011	Esperhohle	Mmyotis	f	ad	4
38	2011	Esperhohle	Mmyotis	f	ad	4
38	2011	Esperhohle	Mmyotis	f	ad	4
38	2011	Esperhohle	Mmyotis	f	ad	4
38	2011	Esperhohle	Mmyotis	f	ad	7
38	2011	Esperhohle	Mmyotis	f	ad	7

38	2011	Esperhohle	Mmyotis	f	ad	14
38	2011	Esperhohle	Mmyotis	m	ad	0
38	2011	Esperhohle	Mmyotis	m	ad	0
38	2011	Esperhohle	Mmyotis	m	ad	0
38	2011	Esperhohle	Mmyotis	m	ad	0
38	2011	Esperhohle	Mmyotis	m	ad	0
38	2011	Esperhohle	Mmyotis	m	ad	0
38	2011	Esperhohle	Mmyotis	m	ad	0
38	2011	Esperhohle	Mmyotis	m	ad	0
38	2011	Esperhohle	Mmyotis	m	ad	0
38	2011	Esperhohle	Mmyotis	m	ad	0
38	2011	Esperhohle	Mmyotis	m	ad	0
38	2011	Esperhohle	Mmyotis	m	ad	0
38	2011	Esperhohle	Mmyotis	m	ad	1
38	2011	Esperhohle	Mmyotis	m	ad	1
38	2011	Esperhohle	Mmyotis	m	ad	1
38	2011	Esperhohle	Mmyotis	m	ad	2
38	2011	Esperhohle	Mmyotis	m	ad	2
38	2011	Esperhohle	Mmyotis	m	ad	2
38	2011	Esperhohle	Mmyotis	m	ad	5
38	2011	Esperhohle	Mmyotis	m	ad	6
38	2011	Esperhohle	Mmyotis	m	ad	13
38	2011	Esperhohle	Mmyotis	f	juv	3
38	2011	Esperhohle	Mmyotis	f	juv	4
38	2011	Esperhohle	Mmyotis	f	juv	5
38	2011	Esperhohle	Mmyotis	f	juv	12
38	2011	Esperhohle	Mmyotis	m	juv	0
38	2011	Esperhohle	Mmyotis	m	juv	0
38	2011	Esperhohle	Mmyotis	m	juv	0
38	2011	Esperhohle	Mmyotis	m	juv	1
38	2011	Esperhohle	Mmyotis	m	juv	2
38	2011	Esperhohle	Mmyotis	m	juv	2
38	2011	Esperhohle	Mmyotis	m	juv	2
38	2011	Esperhohle	Mmyotis	m	juv	3
38	2011	Esperhohle	Mmyotis	m	juv	5
38	2011	Esperhohle	Mnattereri	f	ad	0
38	2011	Esperhohle	Mnattereri	f	ad	1
38	2011	Esperhohle	Mnattereri	m	ad	0
38	2011	Esperhohle	Mnattereri	m	ad	0
38	2011	Esperhohle	Mnattereri	m	ad	0
38	2011	Esperhohle	Mnattereri	f	juv	4

38	2011	Esperhohle	Mnattereri	m	juv	0
38	2011	Esperhohle	Mnattereri	m	juv	0
38	2011	Esperhohle	Mnattereri	m	juv	0
38	2011	Esperhohle	Mnattereri	m	juv	0
38	2011	Esperhohle	Mnattereri	m	juv	1
39	2011	Brunnen Mey	Mdaubenton	f	ad	0
39	2011	Brunnen Mey	Mdaubenton	f	ad	0
39	2011	Brunnen Mey	Mdaubenton	f	ad	1
39	2011	Brunnen Mey	Mdaubenton	f	ad	2
39	2011	Brunnen Mey	Mdaubenton	f	ad	2
39	2011	Brunnen Mey	Mdaubenton	m	ad	0
39	2011	Brunnen Mey	Mdaubenton	m	ad	6
39	2011	Brunnen Mey	Mdaubenton	m	ad	8
39	2011	Brunnen Mey	Mdaubenton	f	juv	0
39	2011	Brunnen Mey	Mdaubenton	f	juv	1
39	2011	Brunnen Mey	Mdaubenton	m	juv	0
39	2011	Brunnen Mey	Mdaubenton	m	juv	1
39	2011	Brunnen Mey	Mnattereri	f	ad	0
39	2011	Brunnen Mey	Mnattereri	f	ad	0
39	2011	Brunnen Mey	Mnattereri	f	ad	0
39	2011	Brunnen Mey	Mnattereri	f	ad	0
39	2011	Brunnen Mey	Mnattereri	f	ad	1
39	2011	Brunnen Mey	Mnattereri	f	ad	1
39	2011	Brunnen Mey	Mnattereri	f	ad	1
39	2011	Brunnen Mey	Mnattereri	f	ad	3
39	2011	Brunnen Mey	Mnattereri	m	ad	0
39	2011	Brunnen Mey	Mnattereri	m	ad	0
39	2011	Brunnen Mey	Mnattereri	m	ad	0
39	2011	Brunnen Mey	Mnattereri	m	ad	0
39	2011	Brunnen Mey	Mnattereri	m	ad	0
39	2011	Brunnen Mey	Mnattereri	m	ad	0
39	2011	Brunnen Mey	Mnattereri	f	juv	0
39	2011	Brunnen Mey	Mnattereri	f	juv	0
39	2011	Brunnen Mey	Mnattereri	f	juv	0
39	2011	Brunnen Mey	Mnattereri	f	juv	0
39	2011	Brunnen Mey	Mnattereri	f	juv	0
39	2011	Brunnen Mey	Mnattereri	f	juv	0
39	2011	Brunnen Mey	Mnattereri	f	juv	1
39	2011	Brunnen Mey	Mnattereri	f	juv	1
39	2011	Brunnen Mey	Mnattereri	f	juv	3
39	2011	Brunnen Mey	Mnattereri	f	juv	4

39	2011	Brunnen Mey	Mnattereri	m	juv	0
39	2011	Brunnen Mey	Mnattereri	m	juv	0
39	2011	Brunnen Mey	Mnattereri	m	juv	0
39	2011	Brunnen Mey	Mnattereri	m	juv	0
39	2011	Brunnen Mey	Mnattereri	m	juv	0
39	2011	Brunnen Mey	Mnattereri	m	juv	0
39	2011	Brunnen Mey	Mnattereri	m	juv	0
39	2011	Brunnen Mey	Mnattereri	m	juv	1
39	2011	Brunnen Mey	Mnattereri	m	juv	1
39	2011	Brunnen Mey	Mnattereri	m	juv	2
39	2011	Brunnen Mey	Mnattereri	m	juv	4
41	2012	Brunnen Mey	Mdaubenton	f	ad	0
41	2012	Brunnen Mey	Mdaubenton	f	ad	1
41	2012	Brunnen Mey	Mdaubenton	f	ad	1
41	2012	Brunnen Mey	Mdaubenton	f	ad	2
41	2012	Brunnen Mey	Mdaubenton	f	ad	3
41	2012	Brunnen Mey	Mdaubenton	m	ad	0
41	2012	Brunnen Mey	Mdaubenton	m	ad	0
41	2012	Brunnen Mey	Mdaubenton	m	ad	1
41	2012	Brunnen Mey	Mdaubenton	m	ad	1
41	2012	Brunnen Mey	Mdaubenton	m	ad	1
41	2012	Brunnen Mey	Mdaubenton	m	ad	2
41	2012	Brunnen Mey	Mdaubenton	m	ad	4
41	2012	Brunnen Mey	Mdaubenton	m	ad	5
41	2012	Brunnen Mey	Mdaubenton	m	ad	5
41	2012	Brunnen Mey	Mdaubenton	f	juv	0
41	2012	Brunnen Mey	Mdaubenton	f	juv	1
41	2012	Brunnen Mey	Mdaubenton	f	juv	2
41	2012	Brunnen Mey	Mdaubenton	m	juv	0
41	2012	Brunnen Mey	Mdaubenton	m	juv	0
41	2012	Brunnen Mey	Mdaubenton	m	juv	8
41	2012	Brunnen Mey	Mmyotis	f	ad	1
41	2012	Brunnen Mey	Mnattereri	f	ad	0
41	2012	Brunnen Mey	Mnattereri	f	ad	0
41	2012	Brunnen Mey	Mnattereri	f	ad	0
41	2012	Brunnen Mey	Mnattereri	f	ad	0
41	2012	Brunnen Mey	Mnattereri	f	ad	0
41	2012	Brunnen Mey	Mnattereri	f	ad	0
41	2012	Brunnen Mey	Mnattereri	f	ad	1
41	2012	Brunnen Mey	Mnattereri	f	ad	1
41	2012	Brunnen Mey	Mnattereri	f	ad	2

41	2012	Brunnen Mey	Mnattereri	f	ad	4
41	2012	Brunnen Mey	Mnattereri	m	ad	0
41	2012	Brunnen Mey	Mnattereri	m	ad	0
41	2012	Brunnen Mey	Mnattereri	m	ad	0
41	2012	Brunnen Mey	Mnattereri	m	ad	0
41	2012	Brunnen Mey	Mnattereri	m	ad	0
41	2012	Brunnen Mey	Mnattereri	m	ad	0
41	2012	Brunnen Mey	Mnattereri	m	ad	0
41	2012	Brunnen Mey	Mnattereri	m	ad	0
41	2012	Brunnen Mey	Mnattereri	f	juv	0
41	2012	Brunnen Mey	Mnattereri	f	juv	0
41	2012	Brunnen Mey	Mnattereri	f	juv	0
41	2012	Brunnen Mey	Mnattereri	f	juv	0
41	2012	Brunnen Mey	Mnattereri	f	juv	0
41	2012	Brunnen Mey	Mnattereri	f	juv	1
41	2012	Brunnen Mey	Mnattereri	f	juv	1
41	2012	Brunnen Mey	Mnattereri	m	juv	0
41	2012	Brunnen Mey	Mnattereri	m	juv	0
41	2012	Brunnen Mey	Mnattereri	m	juv	0
41	2012	Brunnen Mey	Mnattereri	m	juv	0
41	2012	Brunnen Mey	Mnattereri	m	juv	0
41	2012	Brunnen Mey	Mnattereri	m	juv	0
41	2012	Brunnen Mey	Mnattereri	m	juv	0
41	2012	Brunnen Mey	Mnattereri	m	juv	0
41	2012	Brunnen Mey	Mnattereri	m	juv	0
41	2012	Brunnen Mey	Mnattereri	m	juv	0
41	2012	Brunnen Mey	Mnattereri	m	juv	0
41	2012	Brunnen Mey	Mnattereri	m	juv	0
41	2012	Brunnen Mey	Mnattereri	m	juv	0
41	2012	Brunnen Mey	Mnattereri	m	juv	1
41	2012	Brunnen Mey	Mnattereri	m	juv	3
41	2012	Brunnen Mey	Mnattereri	m	juv	3
46	2012	Esperhohle	Mdaubenton	f	ad	1
46	2012	Esperhohle	Mdaubenton	m	ad	0
46	2012	Esperhohle	Mmyotis	f	ad	1
46	2012	Esperhohle	Mmyotis	f	ad	1
46	2012	Esperhohle	Mmyotis	f	ad	2
46	2012	Esperhohle	Mmyotis	f	ad	2
46	2012	Esperhohle	Mmyotis	f	ad	2
46	2012	Esperhohle	Mmyotis	f	ad	3
46	2012	Esperhohle	Mmyotis	f	ad	4

46	2012	Esperhohle	Mmyotis	f	ad	5
46	2012	Esperhohle	Mmyotis	f	ad	5
46	2012	Esperhohle	Mmyotis	f	ad	5
46	2012	Esperhohle	Mmyotis	f	ad	5
46	2012	Esperhohle	Mmyotis	f	ad	7
46	2012	Esperhohle	Mmyotis	f	ad	8
46	2012	Esperhohle	Mmyotis	f	ad	10
46	2012	Esperhohle	Mmyotis	f	ad	11
46	2012	Esperhohle	Mmyotis	m	ad	0
46	2012	Esperhohle	Mmyotis	m	ad	0
46	2012	Esperhohle	Mmyotis	m	ad	0
46	2012	Esperhohle	Mmyotis	m	ad	0
46	2012	Esperhohle	Mmyotis	m	ad	0
46	2012	Esperhohle	Mmyotis	m	ad	0
46	2012	Esperhohle	Mmyotis	m	ad	0
46	2012	Esperhohle	Mmyotis	m	ad	0
46	2012	Esperhohle	Mmyotis	m	ad	0
46	2012	Esperhohle	Mmyotis	m	ad	0
46	2012	Esperhohle	Mmyotis	m	ad	0
46	2012	Esperhohle	Mmyotis	m	ad	0
46	2012	Esperhohle	Mmyotis	m	ad	0
46	2012	Esperhohle	Mmyotis	m	ad	0
46	2012	Esperhohle	Mmyotis	m	ad	0
46	2012	Esperhohle	Mmyotis	m	ad	0
46	2012	Esperhohle	Mmyotis	m	ad	0
46	2012	Esperhohle	Mmyotis	m	ad	0
46	2012	Esperhohle	Mmyotis	m	ad	0
46	2012	Esperhohle	Mmyotis	m	ad	0
46	2012	Esperhohle	Mmyotis	m	ad	0
46	2012	Esperhohle	Mmyotis	m	ad	1
46	2012	Esperhohle	Mmyotis	m	ad	1
46	2012	Esperhohle	Mmyotis	m	ad	1
46	2012	Esperhohle	Mmyotis	m	ad	1
46	2012	Esperhohle	Mmyotis	m	ad	2
46	2012	Esperhohle	Mmyotis	m	ad	2
46	2012	Esperhohle	Mmyotis	m	ad	2
46	2012	Esperhohle	Mmyotis	m	ad	2
46	2012	Esperhohle	Mmyotis	m	ad	2
46	2012	Esperhohle	Mmyotis	m	ad	2
46	2012	Esperhohle	Mmyotis	m	ad	2
46	2012	Esperhohle	Mmyotis	m	ad	3

46	2012	Esperhohle	Mmyotis	m	ad	3
46	2012	Esperhohle	Mmyotis	m	ad	3
46	2012	Esperhohle	Mmyotis	m	ad	4
46	2012	Esperhohle	Mmyotis	m	ad	7
46	2012	Esperhohle	Mmyotis	m	ad	7
46	2012	Esperhohle	Mmyotis	f	juv	0
46	2012	Esperhohle	Mmyotis	f	juv	1
46	2012	Esperhohle	Mmyotis	f	juv	2
46	2012	Esperhohle	Mmyotis	f	juv	2
46	2012	Esperhohle	Mmyotis	f	juv	2
46	2012	Esperhohle	Mmyotis	f	juv	2
46	2012	Esperhohle	Mmyotis	f	juv	2
46	2012	Esperhohle	Mmyotis	f	juv	2
46	2012	Esperhohle	Mmyotis	f	juv	3
46	2012	Esperhohle	Mmyotis	f	juv	3
46	2012	Esperhohle	Mmyotis	f	juv	5
46	2012	Esperhohle	Mmyotis	f	juv	6
46	2012	Esperhohle	Mmyotis	f	juv	6
46	2012	Esperhohle	Mmyotis	f	juv	7
46	2012	Esperhohle	Mmyotis	f	juv	9
46	2012	Esperhohle	Mmyotis	m	juv	0
46	2012	Esperhohle	Mmyotis	m	juv	0
46	2012	Esperhohle	Mmyotis	m	juv	0
46	2012	Esperhohle	Mmyotis	m	juv	0
46	2012	Esperhohle	Mmyotis	m	juv	0
46	2012	Esperhohle	Mmyotis	m	juv	0
46	2012	Esperhohle	Mmyotis	m	juv	0
46	2012	Esperhohle	Mmyotis	m	juv	0
46	2012	Esperhohle	Mmyotis	m	juv	1
46	2012	Esperhohle	Mmyotis	m	juv	1
46	2012	Esperhohle	Mmyotis	m	juv	1
46	2012	Esperhohle	Mmyotis	m	juv	2
46	2012	Esperhohle	Mmyotis	m	juv	2
46	2012	Esperhohle	Mmyotis	m	juv	2
46	2012	Esperhohle	Mmyotis	m	juv	2
46	2012	Esperhohle	Mmyotis	m	juv	2
46	2012	Esperhohle	Mmyotis	m	juv	2
46	2012	Esperhohle	Mmyotis	m	juv	2
46	2012	Esperhohle	Mmyotis	m	juv	3
46	2012	Esperhohle	Mmyotis	m	juv	5
46	2012	Esperhohle	Mmyotis	m	juv	5

46	2012	Esperhohle	Mmyotis	m	juv	5
46	2012	Esperhohle	Mmyotis	m	juv	5
46	2012	Esperhohle	Mmyotis	m	juv	5
46	2012	Esperhohle	Mmyotis	m	juv	5
46	2012	Esperhohle	Mmyotis	m	juv	6
46	2012	Esperhohle	Mmyotis	m	juv	6
46	2012	Esperhohle	Mmyotis	m	juv	12
46	2012	Esperhohle	Mmyotis	m	juv	14
46	2012	Esperhohle	Mmyotis	m	juv	15
46	2012	Esperhohle	Mnattereri	f	ad	0
46	2012	Esperhohle	Mnattereri	f	ad	0
46	2012	Esperhohle	Mnattereri	f	ad	0
46	2012	Esperhohle	Mnattereri	f	ad	1
46	2012	Esperhohle	Mnattereri	m	ad	0
46	2012	Esperhohle	Mnattereri	m	ad	0
46	2012	Esperhohle	Mnattereri	m	ad	0
46	2012	Esperhohle	Mnattereri	m	ad	0
46	2012	Esperhohle	Mnattereri	m	ad	0
46	2012	Esperhohle	Mnattereri	m	ad	0
46	2012	Esperhohle	Mnattereri	m	ad	0
46	2012	Esperhohle	Mnattereri	m	ad	0
46	2012	Esperhohle	Mnattereri	m	ad	0
46	2012	Esperhohle	Mnattereri	m	ad	0
46	2012	Esperhohle	Mnattereri	m	ad	0
46	2012	Esperhohle	Mnattereri	m	ad	0
46	2012	Esperhohle	Mnattereri	f	juv	0
46	2012	Esperhohle	Mnattereri	f	juv	1
46	2012	Esperhohle	Mnattereri	f	juv	5
46	2012	Esperhohle	Mnattereri	m	juv	0
46	2012	Esperhohle	Mnattereri	m	juv	0
46	2012	Esperhohle	Mnattereri	m	juv	0
46	2012	Esperhohle	Mnattereri	m	juv	1
46	2012	Esperhohle	Mnattereri	m	juv	1
46	2012	Esperhohle	Mnattereri	m	juv	4
47	2011	Schonstenhol	Mdaubenton	m	ad	1
47	2011	Schonstenhol	Mdaubenton	m	juv	0
47	2011	Schonstenhol	Mdaubenton	m	juv	1
47	2012	Schonstenhol	Mdaubenton	m	juv	1
47	2011	Schonstenhol	Mmyotis	f	ad	0
47	2012	Schonstenhol	Mmyotis	f	ad	3
47	2011	Schonstenhol	Mmyotis	f	ad	4

47	2012	Schonstenhol	Mmyotis	f	ad	7
47	2011	Schonstenhol	Mmyotis	m	ad	0
47	2011	Schonstenhol	Mmyotis	m	ad	0
47	2011	Schonstenhol	Mmyotis	m	ad	0
47	2011	Schonstenhol	Mmyotis	m	ad	0
47	2012	Schonstenhol	Mmyotis	m	ad	0
47	2011	Schonstenhol	Mmyotis	m	ad	0
47	2011	Schonstenhol	Mmyotis	m	ad	0
47	2012	Schonstenhol	Mmyotis	m	ad	0
47	2012	Schonstenhol	Mmyotis	m	ad	1
47	2011	Schonstenhol	Mmyotis	m	ad	2
47	2011	Schonstenhol	Mmyotis	f	juv	0
47	2011	Schonstenhol	Mmyotis	f	juv	2
47	2011	Schonstenhol	Mmyotis	f	juv	3
47	2011	Schonstenhol	Mmyotis	f	juv	4
47	2011	Schonstenhol	Mmyotis	f	juv	7
47	2012	Schonstenhol	Mmyotis	f	juv	12
47	2012	Schonstenhol	Mmyotis	m	juv	0
47	2012	Schonstenhol	Mmyotis	m	juv	0
47	2012	Schonstenhol	Mmyotis	m	juv	0
47	2012	Schonstenhol	Mmyotis	m	juv	0
47	2011	Schonstenhol	Mmyotis	m	juv	1
47	2011	Schonstenhol	Mmyotis	m	juv	1
47	2012	Schonstenhol	Mmyotis	m	juv	4
47	2012	Schonstenhol	Mmyotis	m	juv	5
47	2011	Schonstenhol	Mnattereri	f	ad	0
47	2012	Schonstenhol	Mnattereri	f	ad	0
47	2011	Schonstenhol	Mnattereri	f	ad	0
47	2012	Schonstenhol	Mnattereri	f	ad	0
47	2012	Schonstenhol	Mnattereri	f	ad	0
47	2012	Schonstenhol	Mnattereri	f	ad	0
47	2012	Schonstenhol	Mnattereri	f	ad	0
47	2012	Schonstenhol	Mnattereri	f	ad	0
47	2012	Schonstenhol	Mnattereri	f	ad	0
47	2011	Schonstenhol	Mnattereri	f	ad	0
47	2011	Schonstenhol	Mnattereri	f	ad	0
47	2011	Schonstenhol	Mnattereri	f	ad	0
47	2012	Schonstenhol	Mnattereri	f	ad	0
47	2012	Schonstenhol	Mnattereri	f	ad	0
47	2011	Schonstenhol	Mnattereri	f	ad	0
47	2012	Schonstenhol	Mnattereri	f	ad	0

47	2011	Schonstenhol	Mnattereri	f	ad	1
47	2012	Schonstenhol	Mnattereri	f	ad	1
47	2012	Schonstenhol	Mnattereri	f	ad	1
47	2011	Schonstenhol	Mnattereri	f	ad	1
47	2012	Schonstenhol	Mnattereri	f	ad	1
47	2012	Schonstenhol	Mnattereri	f	ad	1
47	2012	Schonstenhol	Mnattereri	f	ad	2
47	2012	Schonstenhol	Mnattereri	f	ad	2
47	2012	Schonstenhol	Mnattereri	f	ad	2
47	2011	Schonstenhol	Mnattereri	f	ad	2
47	2012	Schonstenhol	Mnattereri	f	ad	3
47	2011	Schonstenhol	Mnattereri	m	ad	0
47	2012	Schonstenhol	Mnattereri	m	ad	0
47	2012	Schonstenhol	Mnattereri	m	ad	0
47	2012	Schonstenhol	Mnattereri	m	ad	0
47	2012	Schonstenhol	Mnattereri	m	ad	0
47	2012	Schonstenhol	Mnattereri	m	ad	0
47	2012	Schonstenhol	Mnattereri	m	ad	0
47	2011	Schonstenhol	Mnattereri	m	ad	0
47	2012	Schonstenhol	Mnattereri	m	ad	0
47	2012	Schonstenhol	Mnattereri	m	ad	0
47	2012	Schonstenhol	Mnattereri	m	ad	0
47	2012	Schonstenhol	Mnattereri	m	ad	0
47	2012	Schonstenhol	Mnattereri	m	ad	0
47	2012	Schonstenhol	Mnattereri	m	ad	0
47	2011	Schonstenhol	Mnattereri	m	ad	0
47	2012	Schonstenhol	Mnattereri	m	ad	0
47	2012	Schonstenhol	Mnattereri	m	ad	0
47	2012	Schonstenhol	Mnattereri	m	ad	0
47	2012	Schonstenhol	Mnattereri	m	ad	0
47	2011	Schonstenhol	Mnattereri	m	ad	0
47	2012	Schonstenhol	Mnattereri	m	ad	0
47	2012	Schonstenhol	Mnattereri	m	ad	0
47	2012	Schonstenhol	Mnattereri	m	ad	0
47	2012	Schonstenhol	Mnattereri	m	ad	0
47	2012	Schonstenhol	Mnattereri	m	ad	0
47	2012	Schonstenhol	Mnattereri	m	ad	0
47	2011	Schonstenhol	Mnattereri	m	ad	0
47	2011	Schonstenhol	Mnattereri	m	ad	0
47	2012	Schonstenhol	Mnattereri	m	ad	0
47	2012	Schonstenhol	Mnattereri	m	ad	0

47	2012	Schonstenhol	Mnattereri	m	ad	0
47	2012	Schonstenhol	Mnattereri	m	ad	0
47	2011	Schonstenhol	Mnattereri	m	ad	0
47	2012	Schonstenhol	Mnattereri	m	ad	0
47	2011	Schonstenhol	Mnattereri	m	ad	0
47	2011	Schonstenhol	Mnattereri	m	ad	0
47	2011	Schonstenhol	Mnattereri	m	ad	0
47	2011	Schonstenhol	Mnattereri	m	ad	0
47	2011	Schonstenhol	Mnattereri	m	ad	0
47	2011	Schonstenhol	Mnattereri	m	ad	0
47	2011	Schonstenhol	Mnattereri	m	ad	0
47	2011	Schonstenhol	Mnattereri	m	ad	0
47	2011	Schonstenhol	Mnattereri	m	ad	0
47	2011	Schonstenhol	Mnattereri	m	ad	0
47	2011	Schonstenhol	Mnattereri	m	ad	0
47	2011	Schonstenhol	Mnattereri	m	ad	0
47	2011	Schonstenhol	Mnattereri	m	ad	0
47	2011	Schonstenhol	Mnattereri	m	ad	0
47	2011	Schonstenhol	Mnattereri	m	ad	0
47	2011	Schonstenhol	Mnattereri	m	ad	0
47	2011	Schonstenhol	Mnattereri	m	ad	0
47	2011	Schonstenhol	Mnattereri	m	ad	0
47	2011	Schonstenhol	Mnattereri	m	ad	0
47	2011	Schonstenhol	Mnattereri	m	ad	0
47	2011	Schonstenhol	Mnattereri	m	ad	0
47	2011	Schonstenhol	Mnattereri	m	ad	0
47	2011	Schonstenhol	Mnattereri	m	ad	0
47	2012	Schonstenhol	Mnattereri	m	ad	0
47	2012	Schonstenhol	Mnattereri	m	ad	0
47	2012	Schonstenhol	Mnattereri	m	ad	0
47	2012	Schonstenhol	Mnattereri	m	ad	0
47	2012	Schonstenhol	Mnattereri	m	ad	0
47	2012	Schonstenhol	Mnattereri	m	ad	0
47	2012	Schonstenhol	Mnattereri	m	ad	0
47	2012	Schonstenhol	Mnattereri	m	ad	0
47	2012	Schonstenhol	Mnattereri	m	ad	0
47	2012	Schonstenhol	Mnattereri	m	ad	0
47	2012	Schonstenhol	Mnattereri	m	ad	0
47	2012	Schonstenhol	Mnattereri	m	ad	0
47	2012	Schonstenhol	Mnattereri	m	ad	0
47	2011	Schonstenhol	Mnattereri	m	ad	0

47	2012	Schonstenhol	Mnattereri	m	ad	4
47	2012	Schonstenhol	Mnattereri	f	juv	0
47	2012	Schonstenhol	Mnattereri	f	juv	0
47	2012	Schonstenhol	Mnattereri	f	juv	0
47	2012	Schonstenhol	Mnattereri	f	juv	0
47	2012	Schonstenhol	Mnattereri	f	juv	0
47	2011	Schonstenhol	Mnattereri	f	juv	1
47	2012	Schonstenhol	Mnattereri	f	juv	1
47	2012	Schonstenhol	Mnattereri	f	juv	3
47	2012	Schonstenhol	Mnattereri	m	juv	0
47	2011	Schonstenhol	Mnattereri	m	juv	0
47	2012	Schonstenhol	Mnattereri	m	juv	0
47	2012	Schonstenhol	Mnattereri	m	juv	0
47	2012	Schonstenhol	Mnattereri	m	juv	0
47	2011	Schonstenhol	Mnattereri	m	juv	0
47	2012	Schonstenhol	Mnattereri	m	juv	0
47	2012	Schonstenhol	Mnattereri	m	juv	0
47	2012	Schonstenhol	Mnattereri	m	juv	0
47	2011	Schonstenhol	Mnattereri	m	juv	0
47	2011	Schonstenhol	Mnattereri	m	juv	0
47	2012	Schonstenhol	Mnattereri	m	juv	0
47	2012	Schonstenhol	Mnattereri	m	juv	0
47	2012	Schonstenhol	Mnattereri	m	juv	0
47	2012	Schonstenhol	Mnattereri	m	juv	0
47	2012	Schonstenhol	Mnattereri	m	juv	0
47	2012	Schonstenhol	Mnattereri	m	juv	0
47	2012	Schonstenhol	Mnattereri	m	juv	0
47	2012	Schonstenhol	Mnattereri	m	juv	0
47	2012	Schonstenhol	Mnattereri	m	juv	0
47	2012	Schonstenhol	Mnattereri	m	juv	0
47	2012	Schonstenhol	Mnattereri	m	juv	0
47	2012	Schonstenhol	Mnattereri	m	juv	0
47	2011	Schonstenhol	Mnattereri	m	juv	0
47	2012	Schonstenhol	Mnattereri	m	juv	1
47	2011	Schonstenhol	Mnattereri	m	juv	1
47	2012	Schonstenhol	Mnattereri	m	juv	2
47	2012	Schonstenhol	Mnattereri	m	juv	2
47	2012	Schonstenhol	Mnattereri	m	juv	3
47	2012	Schonstenhol	Mnattereri	m	juv	4
48	2011	Esperhohle	Mmyotis	f	ad	0
48	2011	Esperhohle	Mmyotis	f	ad	0

48	2011	Esperhohle	Mmyotis	f	ad	0
48	2011	Esperhohle	Mmyotis	f	ad	2
48	2011	Esperhohle	Mmyotis	f	ad	3
48	2011	Esperhohle	Mmyotis	f	ad	5
48	2011	Esperhohle	Mmyotis	m	ad	0
48	2011	Esperhohle	Mmyotis	m	ad	0
48	2011	Esperhohle	Mmyotis	m	ad	0
48	2011	Esperhohle	Mmyotis	m	ad	0
48	2011	Esperhohle	Mmyotis	m	ad	0
48	2011	Esperhohle	Mmyotis	m	ad	0
48	2011	Esperhohle	Mmyotis	m	ad	0
48	2011	Esperhohle	Mmyotis	m	ad	0
48	2011	Esperhohle	Mmyotis	m	ad	0
48	2011	Esperhohle	Mmyotis	m	ad	0
48	2011	Esperhohle	Mmyotis	m	ad	0
48	2011	Esperhohle	Mmyotis	m	ad	0
48	2011	Esperhohle	Mmyotis	m	ad	0
48	2011	Esperhohle	Mmyotis	m	ad	1
48	2011	Esperhohle	Mmyotis	m	ad	1
48	2011	Esperhohle	Mmyotis	m	ad	1
48	2011	Esperhohle	Mmyotis	m	ad	1
48	2011	Esperhohle	Mmyotis	m	ad	1
48	2011	Esperhohle	Mmyotis	m	ad	2
48	2011	Esperhohle	Mmyotis	m	ad	2
48	2011	Esperhohle	Mmyotis	m	ad	2
48	2011	Esperhohle	Mmyotis	m	ad	3
48	2011	Esperhohle	Mmyotis	m	ad	6
48	2011	Esperhohle	Mmyotis	f	juv	3
48	2011	Esperhohle	Mmyotis	m	juv	0
48	2011	Esperhohle	Mmyotis	m	juv	0
48	2011	Esperhohle	Mmyotis	m	juv	1
50	2011	Brunnen Mey	Mdaubenton	f	ad	0
50	2011	Brunnen Mey	Mdaubenton	f	ad	0
50	2011	Brunnen Mey	Mdaubenton	f	ad	0
50	2012	Brunnen Mey	Mdaubenton	f	ad	0
50	2011	Brunnen Mey	Mdaubenton	f	ad	1
50	2012	Brunnen Mey	Mdaubenton	f	ad	1
50	2011	Brunnen Mey	Mdaubenton	f	ad	2
50	2011	Brunnen Mey	Mdaubenton	f	ad	3
50	2011	Brunnen Mey	Mdaubenton	f	ad	4
50	2011	Brunnen Mey	Mdaubenton	m	ad	0

50	2011	Brunnen Mey	Mdaubenton	m	ad	1
50	2012	Brunnen Mey	Mdaubenton	m	ad	1
50	2011	Brunnen Mey	Mdaubenton	m	ad	1
50	2012	Brunnen Mey	Mdaubenton	m	ad	1
50	2011	Brunnen Mey	Mdaubenton	f	juv	0
50	2011	Brunnen Mey	Mdaubenton	f	juv	0
50	2012	Brunnen Mey	Mdaubenton	f	juv	0
50	2012	Brunnen Mey	Mdaubenton	f	juv	0
50	2012	Brunnen Mey	Mdaubenton	f	juv	0
50	2011	Brunnen Mey	Mdaubenton	f	juv	1
50	2012	Brunnen Mey	Mdaubenton	f	juv	1
50	2012	Brunnen Mey	Mdaubenton	f	juv	2
50	2011	Brunnen Mey	Mdaubenton	m	juv	0
50	2011	Brunnen Mey	Mdaubenton	m	juv	0
50	2012	Brunnen Mey	Mdaubenton	m	juv	0
50	2012	Brunnen Mey	Mdaubenton	m	juv	0
50	2012	Brunnen Mey	Mmyotis	m	juv	0
50	2011	Brunnen Mey	Mnattereri	f	ad	0
50	2011	Brunnen Mey	Mnattereri	f	ad	0
50	2012	Brunnen Mey	Mnattereri	f	ad	0
50	2012	Brunnen Mey	Mnattereri	f	ad	0
50	2012	Brunnen Mey	Mnattereri	f	ad	0
50	2012	Brunnen Mey	Mnattereri	f	ad	0
50	2012	Brunnen Mey	Mnattereri	f	ad	0
50	2012	Brunnen Mey	Mnattereri	f	ad	0
50	2012	Brunnen Mey	Mnattereri	f	ad	0
50	2012	Brunnen Mey	Mnattereri	f	ad	0
50	2012	Brunnen Mey	Mnattereri	f	ad	1
50	2012	Brunnen Mey	Mnattereri	f	ad	1
50	2012	Brunnen Mey	Mnattereri	f	ad	1
50	2011	Brunnen Mey	Mnattereri	f	ad	7
50	2011	Brunnen Mey	Mnattereri	m	ad	0
50	2011	Brunnen Mey	Mnattereri	m	ad	0
50	2011	Brunnen Mey	Mnattereri	m	ad	0
50	2011	Brunnen Mey	Mnattereri	m	ad	0
50	2011	Brunnen Mey	Mnattereri	m	ad	0
50	2011	Brunnen Mey	Mnattereri	m	ad	0
50	2011	Brunnen Mey	Mnattereri	m	ad	0
50	2011	Brunnen Mey	Mnattereri	m	ad	0
50	2011	Brunnen Mey	Mnattereri	m	ad	0
50	2011	Brunnen Mey	Mnattereri	m	ad	0

50	2011	Brunnen Mey	Mnattereri	m	ad	0
50	2012	Brunnen Mey	Mnattereri	m	ad	0
50	2012	Brunnen Mey	Mnattereri	m	ad	0
50	2012	Brunnen Mey	Mnattereri	m	ad	0
50	2012	Brunnen Mey	Mnattereri	m	ad	0
50	2012	Brunnen Mey	Mnattereri	m	ad	0
50	2012	Brunnen Mey	Mnattereri	m	ad	0
50	2012	Brunnen Mey	Mnattereri	m	ad	0
50	2012	Brunnen Mey	Mnattereri	m	ad	0
50	2012	Brunnen Mey	Mnattereri	m	ad	0
50	2012	Brunnen Mey	Mnattereri	m	ad	0
50	2012	Brunnen Mey	Mnattereri	m	ad	0
50	2012	Brunnen Mey	Mnattereri	m	ad	0
50	2012	Brunnen Mey	Mnattereri	m	ad	0
50	2012	Brunnen Mey	Mnattereri	m	ad	0
50	2012	Brunnen Mey	Mnattereri	m	ad	0
50	2012	Brunnen Mey	Mnattereri	m	ad	1
50	2012	Brunnen Mey	Mnattereri	m	ad	1
50	2011	Brunnen Mey	Mnattereri	m	ad	1
50	2011	Brunnen Mey	Mnattereri	f	juv	0
50	2011	Brunnen Mey	Mnattereri	f	juv	0
50	2011	Brunnen Mey	Mnattereri	f	juv	0
50	2011	Brunnen Mey	Mnattereri	f	juv	0
50	2011	Brunnen Mey	Mnattereri	f	juv	0
50	2011	Brunnen Mey	Mnattereri	f	juv	0
50	2011	Brunnen Mey	Mnattereri	f	juv	0
50	2012	Brunnen Mey	Mnattereri	f	juv	0
50	2012	Brunnen Mey	Mnattereri	f	juv	0
50	2012	Brunnen Mey	Mnattereri	f	juv	0
50	2012	Brunnen Mey	Mnattereri	f	juv	0
50	2012	Brunnen Mey	Mnattereri	f	juv	0
50	2012	Brunnen Mey	Mnattereri	f	juv	1
50	2012	Brunnen Mey	Mnattereri	f	juv	1
50	2012	Brunnen Mey	Mnattereri	f	juv	1
50	2011	Brunnen Mey	Mnattereri	f	juv	2
50	2012	Brunnen Mey	Mnattereri	f	juv	3
50	2012	Brunnen Mey	Mnattereri	f	juv	6
50	2011	Brunnen Mey	Mnattereri	m	juv	0
50	2011	Brunnen Mey	Mnattereri	m	juv	0
50	2011	Brunnen Mey	Mnattereri	m	juv	0
50	2011	Brunnen Mey	Mnattereri	m	juv	0

50	2011	Brunnen Mey	Mnattereri	m	juv	0
50	2011	Brunnen Mey	Mnattereri	m	juv	0
50	2011	Brunnen Mey	Mnattereri	m	juv	0
50	2011	Brunnen Mey	Mnattereri	m	juv	0
50	2011	Brunnen Mey	Mnattereri	m	juv	0
50	2011	Brunnen Mey	Mnattereri	m	juv	0
50	2011	Brunnen Mey	Mnattereri	m	juv	0
50	2011	Brunnen Mey	Mnattereri	m	juv	0
50	2011	Brunnen Mey	Mnattereri	m	juv	0
50	2011	Brunnen Mey	Mnattereri	m	juv	0
50	2011	Brunnen Mey	Mnattereri	m	juv	0
50	2011	Brunnen Mey	Mnattereri	m	juv	0
50	2011	Brunnen Mey	Mnattereri	m	juv	0
50	2012	Brunnen Mey	Mnattereri	m	juv	0
50	2012	Brunnen Mey	Mnattereri	m	juv	0
50	2012	Brunnen Mey	Mnattereri	m	juv	0
50	2012	Brunnen Mey	Mnattereri	m	juv	0
50	2012	Brunnen Mey	Mnattereri	m	juv	0
50	2012	Brunnen Mey	Mnattereri	m	juv	0
50	2012	Brunnen Mey	Mnattereri	m	juv	0
50	2012	Brunnen Mey	Mnattereri	m	juv	0
50	2012	Brunnen Mey	Mnattereri	m	juv	0
50	2012	Brunnen Mey	Mnattereri	m	juv	0
50	2012	Brunnen Mey	Mnattereri	m	juv	0
50	2012	Brunnen Mey	Mnattereri	m	juv	0
50	2011	Brunnen Mey	Mnattereri	m	juv	1
50	2011	Brunnen Mey	Mnattereri	m	juv	1
50	2011	Brunnen Mey	Mnattereri	m	juv	1
50	2011	Brunnen Mey	Mnattereri	m	juv	1
50	2012	Brunnen Mey	Mnattereri	m	juv	1
50	2012	Brunnen Mey	Mnattereri	m	juv	1
50	2012	Brunnen Mey	Mnattereri	m	juv	1
50	2012	Brunnen Mey	Mnattereri	m	juv	1
50	2012	Brunnen Mey	Mnattereri	m	juv	1
50	2012	Brunnen Mey	Mnattereri	m	juv	1
50	2011	Brunnen Mey	Mnattereri	m	juv	3
57	2012	Schonstenhol	Mdaubenton	f	juv	0
57	2011	Schonstenhol	Mdaubenton	m	juv	0
57	2011	Schonstenhol	Mdaubenton	m	juv	1
57	2011	Schonstenhol	Mmyotis	f	ad	4
57	2012	Schonstenhol	Mmyotis	f	ad	8

57	2011	Schonstenhol	Mmyotis	m	ad	0
57	2011	Schonstenhol	Mmyotis	m	ad	0
57	2011	Schonstenhol	Mmyotis	m	ad	0
57	2011	Schonstenhol	Mmyotis	m	ad	4
57	2011	Schonstenhol	Mmyotis	f	juv	0
57	2011	Schonstenhol	Mmyotis	f	juv	0
57	2011	Schonstenhol	Mmyotis	f	juv	1
57	2011	Schonstenhol	Mmyotis	m	juv	2
57	2011	Schonstenhol	Mmyotis	m	juv	2
57	2011	Schonstenhol	Mnattereri	f	ad	0
57	2011	Schonstenhol	Mnattereri	f	ad	0
57	2011	Schonstenhol	Mnattereri	f	ad	0
57	2011	Schonstenhol	Mnattereri	f	ad	0
57	2011	Schonstenhol	Mnattereri	f	ad	0
57	2011	Schonstenhol	Mnattereri	f	ad	0
57	2011	Schonstenhol	Mnattereri	f	ad	0
57	2011	Schonstenhol	Mnattereri	f	ad	0
57	2011	Schonstenhol	Mnattereri	f	ad	0
57	2011	Schonstenhol	Mnattereri	f	ad	0
57	2011	Schonstenhol	Mnattereri	f	ad	0
57	2011	Schonstenhol	Mnattereri	f	ad	0
57	2011	Schonstenhol	Mnattereri	f	ad	0
57	2012	Schonstenhol	Mnattereri	f	ad	0
57	2011	Schonstenhol	Mnattereri	f	ad	0
57	2011	Schonstenhol	Mnattereri	f	ad	0
57	2011	Schonstenhol	Mnattereri	f	ad	0
57	2011	Schonstenhol	Mnattereri	f	ad	0
57	2011	Schonstenhol	Mnattereri	f	ad	0
57	2011	Schonstenhol	Mnattereri	f	ad	0
57	2011	Schonstenhol	Mnattereri	f	ad	0
57	2011	Schonstenhol	Mnattereri	f	ad	0
57	2011	Schonstenhol	Mnattereri	f	ad	0
57	2011	Schonstenhol	Mnattereri	f	ad	0
57	2011	Schonstenhol	Mnattereri	f	ad	0
57	2011	Schonstenhol	Mnattereri	f	ad	0
57	2011	Schonstenhol	Mnattereri	f	ad	0
57	2011	Schonstenhol	Mnattereri	f	ad	1
57	2011	Schonstenhol	Mnattereri	f	ad	2
57	2011	Schonstenhol	Mnattereri	m	ad	0
57	2011	Schonstenhol	Mnattereri	m	ad	0
57	2011	Schonstenhol	Mnattereri	m	ad	0

57	2011	Schonstenhol	Mnattereri	m	ad	0
57	2011	Schonstenhol	Mnattereri	m	ad	0
57	2011	Schonstenhol	Mnattereri	m	ad	0
57	2011	Schonstenhol	Mnattereri	m	ad	0
57	2011	Schonstenhol	Mnattereri	m	ad	0
57	2011	Schonstenhol	Mnattereri	m	ad	0
57	2012	Schonstenhol	Mnattereri	m	ad	0
57	2011	Schonstenhol	Mnattereri	m	ad	0
57	2011	Schonstenhol	Mnattereri	m	ad	0
57	2011	Schonstenhol	Mnattereri	m	ad	0
57	2011	Schonstenhol	Mnattereri	m	ad	0
57	2011	Schonstenhol	Mnattereri	m	ad	0
57	2011	Schonstenhol	Mnattereri	m	ad	0
57	2011	Schonstenhol	Mnattereri	m	ad	0
57	2011	Schonstenhol	Mnattereri	m	ad	0
57	2011	Schonstenhol	Mnattereri	m	ad	0
57	2011	Schonstenhol	Mnattereri	m	ad	0
57	2011	Schonstenhol	Mnattereri	m	ad	0
57	2011	Schonstenhol	Mnattereri	m	ad	0
57	2011	Schonstenhol	Mnattereri	m	ad	0
57	2012	Schonstenhol	Mnattereri	m	ad	0
57	2011	Schonstenhol	Mnattereri	m	ad	0
57	2011	Schonstenhol	Mnattereri	m	ad	0
57	2011	Schonstenhol	Mnattereri	m	ad	0
57	2011	Schonstenhol	Mnattereri	m	ad	0
57	2011	Schonstenhol	Mnattereri	m	ad	0
57	2011	Schonstenhol	Mnattereri	m	ad	0
57	2011	Schonstenhol	Mnattereri	m	ad	0
57	2011	Schonstenhol	Mnattereri	m	ad	0
57	2011	Schonstenhol	Mnattereri	m	ad	0
57	2011	Schonstenhol	Mnattereri	m	ad	0
57	2011	Schonstenhol	Mnattereri	m	ad	0
57	2011	Schonstenhol	Mnattereri	m	ad	1
57	2011	Schonstenhol	Mnattereri	m	ad	1
57	2011	Schonstenhol	Mnattereri	m	ad	1
57	2011	Schonstenhol	Mnattereri	m	ad	2
57	2011	Schonstenhol	Mnattereri	f	juv	0
57	2011	Schonstenhol	Mnattereri	f	juv	0
57	2011	Schonstenhol	Mnattereri	f	juv	0
57	2011	Schonstenhol	Mnattereri	f	juv	0
57	2011	Schonstenhol	Mnattereri	f	juv	0

57	2011	Schonstenhol	Mnattereri	f	juv	0
57	2011	Schonstenhol	Mnattereri	f	juv	0
57	2011	Schonstenhol	Mnattereri	f	juv	0
57	2011	Schonstenhol	Mnattereri	f	juv	0
57	2011	Schonstenhol	Mnattereri	f	juv	1
57	2011	Schonstenhol	Mnattereri	m	juv	0
57	2011	Schonstenhol	Mnattereri	m	juv	0
57	2011	Schonstenhol	Mnattereri	m	juv	0
57	2011	Schonstenhol	Mnattereri	m	juv	0
57	2011	Schonstenhol	Mnattereri	m	juv	0
57	2011	Schonstenhol	Mnattereri	m	juv	0
57	2011	Schonstenhol	Mnattereri	m	juv	0
57	2011	Schonstenhol	Mnattereri	m	juv	0
57	2011	Schonstenhol	Mnattereri	m	juv	0
57	2011	Schonstenhol	Mnattereri	m	juv	0
57	2011	Schonstenhol	Mnattereri	m	juv	0
57	2011	Schonstenhol	Mnattereri	m	juv	0
57	2011	Schonstenhol	Mnattereri	m	juv	0
57	2012	Schonstenhol	Mnattereri	m	juv	0
57	2011	Schonstenhol	Mnattereri	m	juv	1
57	2011	Schonstenhol	Mnattereri	m	juv	1
58	2011	Esperhohle	Mdaubenton	f	ad	1
58	2011	Esperhohle	Mmyotis	f	ad	0
58	2011	Esperhohle	Mmyotis	f	ad	0
58	2011	Esperhohle	Mmyotis	f	ad	0
58	2011	Esperhohle	Mmyotis	f	ad	1
58	2011	Esperhohle	Mmyotis	f	ad	1
58	2011	Esperhohle	Mmyotis	f	ad	2
58	2011	Esperhohle	Mmyotis	f	ad	2
58	2011	Esperhohle	Mmyotis	f	ad	2
58	2011	Esperhohle	Mmyotis	f	ad	2
58	2011	Esperhohle	Mmyotis	f	ad	2
58	2011	Esperhohle	Mmyotis	f	ad	3
58	2011	Esperhohle	Mmyotis	f	ad	3
58	2011	Esperhohle	Mmyotis	f	ad	4
58	2011	Esperhohle	Mmyotis	f	ad	5
58	2011	Esperhohle	Mmyotis	f	ad	8
58	2011	Esperhohle	Mmyotis	f	ad	9
58	2011	Esperhohle	Mmyotis	f	ad	12
58	2011	Esperhohle	Mmyotis	m	ad	0
58	2011	Esperhohle	Mmyotis	m	ad	0

58	2011	Esperhohle	Mmyotis	m	ad	0
58	2011	Esperhohle	Mmyotis	m	ad	0
58	2011	Esperhohle	Mmyotis	m	ad	1
58	2011	Esperhohle	Mmyotis	m	ad	1
58	2011	Esperhohle	Mmyotis	m	ad	1
58	2011	Esperhohle	Mmyotis	m	ad	2
58	2011	Esperhohle	Mmyotis	m	ad	3
58	2011	Esperhohle	Mmyotis	m	ad	4
58	2011	Esperhohle	Mmyotis	m	ad	6
58	2011	Esperhohle	Mmyotis	m	ad	6
58	2011	Esperhohle	Mmyotis	f	juv	0
58	2011	Esperhohle	Mmyotis	f	juv	0
58	2011	Esperhohle	Mmyotis	f	juv	0
58	2011	Esperhohle	Mmyotis	f	juv	1
58	2011	Esperhohle	Mmyotis	f	juv	1
58	2011	Esperhohle	Mmyotis	f	juv	1
58	2011	Esperhohle	Mmyotis	f	juv	4
58	2011	Esperhohle	Mmyotis	f	juv	4
58	2011	Esperhohle	Mmyotis	f	juv	4
58	2011	Esperhohle	Mmyotis	f	juv	5
58	2011	Esperhohle	Mmyotis	f	juv	6
58	2011	Esperhohle	Mmyotis	f	juv	6
58	2011	Esperhohle	Mmyotis	f	juv	8
58	2011	Esperhohle	Mmyotis	f	juv	14
58	2011	Esperhohle	Mmyotis	m	juv	0
58	2011	Esperhohle	Mmyotis	m	juv	0
58	2011	Esperhohle	Mmyotis	m	juv	0
58	2011	Esperhohle	Mmyotis	m	juv	0
58	2011	Esperhohle	Mmyotis	m	juv	0
58	2011	Esperhohle	Mmyotis	m	juv	0
58	2011	Esperhohle	Mmyotis	m	juv	0
58	2011	Esperhohle	Mmyotis	m	juv	0
58	2011	Esperhohle	Mmyotis	m	juv	0
58	2011	Esperhohle	Mmyotis	m	juv	0
58	2011	Esperhohle	Mmyotis	m	juv	1
58	2011	Esperhohle	Mmyotis	m	juv	1
58	2011	Esperhohle	Mmyotis	m	juv	2
58	2011	Esperhohle	Mmyotis	m	juv	2
58	2011	Esperhohle	Mmyotis	m	juv	2
58	2011	Esperhohle	Mmyotis	m	juv	2
58	2011	Esperhohle	Mmyotis	m	juv	2

58	2011	Esperhohle	Mmyotis	m	juv	3
58	2011	Esperhohle	Mmyotis	m	juv	4
58	2011	Esperhohle	Mmyotis	m	juv	4
58	2011	Esperhohle	Mmyotis	m	juv	4
58	2011	Esperhohle	Mmyotis	m	juv	6
58	2011	Esperhohle	Mnattereri	f	ad	0
58	2011	Esperhohle	Mnattereri	f	ad	0
58	2011	Esperhohle	Mnattereri	f	ad	0
58	2011	Esperhohle	Mnattereri	f	ad	2
58	2011	Esperhohle	Mnattereri	m	ad	0
58	2011	Esperhohle	Mnattereri	m	ad	0
58	2011	Esperhohle	Mnattereri	m	ad	0
58	2011	Esperhohle	Mnattereri	m	ad	0
58	2011	Esperhohle	Mnattereri	m	ad	0
58	2011	Esperhohle	Mnattereri	m	ad	0
58	2011	Esperhohle	Mnattereri	m	ad	0
58	2011	Esperhohle	Mnattereri	m	ad	0
58	2011	Esperhohle	Mnattereri	m	ad	0
58	2011	Esperhohle	Mnattereri	m	juv	0
58	2011	Esperhohle	Mnattereri	m	juv	0
58	2011	Esperhohle	Mnattereri	m	juv	0
58	2011	Esperhohle	Mnattereri	m	juv	0
58	2011	Esperhohle	Mnattereri	m	juv	0
60	2011	Brunnen Mey	Mdaubenton	f	ad	0
60	2011	Brunnen Mey	Mdaubenton	f	ad	0
60	2011	Brunnen Mey	Mdaubenton	f	ad	0
60	2011	Brunnen Mey	Mdaubenton	f	ad	0
60	2011	Brunnen Mey	Mdaubenton	f	ad	0
60	2011	Brunnen Mey	Mdaubenton	f	ad	0
60	2011	Brunnen Mey	Mdaubenton	f	ad	1
60	2011	Brunnen Mey	Mdaubenton	f	ad	1
60	2011	Brunnen Mey	Mdaubenton	f	ad	2
60	2011	Brunnen Mey	Mdaubenton	f	ad	3
60	2011	Brunnen Mey	Mdaubenton	m	ad	0
60	2011	Brunnen Mey	Mdaubenton	m	ad	0
60	2011	Brunnen Mey	Mdaubenton	m	ad	0
60	2011	Brunnen Mey	Mdaubenton	m	ad	0
60	2011	Brunnen Mey	Mdaubenton	m	ad	0
60	2011	Brunnen Mey	Mdaubenton	m	ad	0
60	2011	Brunnen Mey	Mdaubenton	m	ad	0
60	2011	Brunnen Mey	Mdaubenton	m	ad	0

60	2011	Brunnen Mey	Mdaubenton	m	ad	0
60	2011	Brunnen Mey	Mdaubenton	m	ad	1
60	2011	Brunnen Mey	Mdaubenton	m	ad	2
60	2011	Brunnen Mey	Mdaubenton	m	ad	2
60	2011	Brunnen Mey	Mdaubenton	m	ad	4
60	2011	Brunnen Mey	Mdaubenton	f	juv	0
60	2011	Brunnen Mey	Mdaubenton	f	juv	0
60	2011	Brunnen Mey	Mdaubenton	f	juv	0
60	2011	Brunnen Mey	Mdaubenton	f	juv	0
60	2011	Brunnen Mey	Mdaubenton	f	juv	1
60	2011	Brunnen Mey	Mdaubenton	m	juv	0
60	2011	Brunnen Mey	Mdaubenton	m	juv	0
60	2011	Brunnen Mey	Mdaubenton	m	juv	0
60	2011	Brunnen Mey	Mdaubenton	m	juv	0
60	2011	Brunnen Mey	Mdaubenton	m	juv	0
60	2011	Brunnen Mey	Mdaubenton	m	juv	0
60	2011	Brunnen Mey	Mdaubenton	m	juv	0
60	2011	Brunnen Mey	Mdaubenton	m	juv	2
60	2011	Brunnen Mey	Mnattereri	f	ad	0
60	2011	Brunnen Mey	Mnattereri	f	ad	0
60	2011	Brunnen Mey	Mnattereri	f	ad	0
60	2011	Brunnen Mey	Mnattereri	f	ad	0
60	2011	Brunnen Mey	Mnattereri	f	ad	0
60	2011	Brunnen Mey	Mnattereri	f	ad	0
60	2011	Brunnen Mey	Mnattereri	f	ad	0
60	2011	Brunnen Mey	Mnattereri	f	ad	0
60	2011	Brunnen Mey	Mnattereri	f	ad	1
60	2011	Brunnen Mey	Mnattereri	f	ad	1
60	2011	Brunnen Mey	Mnattereri	f	ad	3
60	2011	Brunnen Mey	Mnattereri	m	ad	0
60	2011	Brunnen Mey	Mnattereri	m	ad	0
60	2011	Brunnen Mey	Mnattereri	m	ad	0
60	2011	Brunnen Mey	Mnattereri	m	ad	0
60	2011	Brunnen Mey	Mnattereri	m	ad	0
60	2011	Brunnen Mey	Mnattereri	m	ad	0
60	2011	Brunnen Mey	Mnattereri	m	ad	0
60	2011	Brunnen Mey	Mnattereri	m	ad	0
60	2011	Brunnen Mey	Mnattereri	m	ad	0
60	2011	Brunnen Mey	Mnattereri	m	ad	0
60	2011	Brunnen Mey	Mnattereri	m	ad	0
60	2011	Brunnen Mey	Mnattereri	m	ad	0

60	2011	Brunnen Mey	Mnattereri	m	ad	0
60	2011	Brunnen Mey	Mnattereri	m	ad	0
60	2011	Brunnen Mey	Mnattereri	m	ad	0
60	2011	Brunnen Mey	Mnattereri	m	ad	0
60	2011	Brunnen Mey	Mnattereri	m	ad	0
60	2011	Brunnen Mey	Mnattereri	m	ad	0
60	2011	Brunnen Mey	Mnattereri	m	ad	0
60	2011	Brunnen Mey	Mnattereri	m	ad	0
60	2011	Brunnen Mey	Mnattereri	m	ad	0
60	2011	Brunnen Mey	Mnattereri	m	ad	0
60	2011	Brunnen Mey	Mnattereri	m	ad	0
60	2011	Brunnen Mey	Mnattereri	m	ad	0
60	2011	Brunnen Mey	Mnattereri	m	ad	0
60	2011	Brunnen Mey	Mnattereri	m	ad	0
60	2011	Brunnen Mey	Mnattereri	m	ad	0
60	2011	Brunnen Mey	Mnattereri	m	ad	0
60	2011	Brunnen Mey	Mnattereri	f	juv	0
60	2011	Brunnen Mey	Mnattereri	f	juv	0
60	2011	Brunnen Mey	Mnattereri	f	juv	0
60	2011	Brunnen Mey	Mnattereri	f	juv	0
60	2011	Brunnen Mey	Mnattereri	f	juv	0
60	2011	Brunnen Mey	Mnattereri	f	juv	0
60	2011	Brunnen Mey	Mnattereri	f	juv	0
60	2011	Brunnen Mey	Mnattereri	f	juv	0
60	2011	Brunnen Mey	Mnattereri	f	juv	0
60	2011	Brunnen Mey	Mnattereri	f	juv	0
60	2011	Brunnen Mey	Mnattereri	f	juv	0
60	2011	Brunnen Mey	Mnattereri	f	juv	0
60	2011	Brunnen Mey	Mnattereri	f	juv	0
60	2011	Brunnen Mey	Mnattereri	f	juv	0
60	2011	Brunnen Mey	Mnattereri	f	juv	1
60	2011	Brunnen Mey	Mnattereri	f	juv	1
60	2011	Brunnen Mey	Mnattereri	f	juv	1
60	2011	Brunnen Mey	Mnattereri	f	juv	2
60	2011	Brunnen Mey	Mnattereri	m	juv	0
60	2011	Brunnen Mey	Mnattereri	m	juv	0
60	2011	Brunnen Mey	Mnattereri	m	juv	0
60	2011	Brunnen Mey	Mnattereri	m	juv	0
60	2011	Brunnen Mey	Mnattereri	m	juv	0
60	2011	Brunnen Mey	Mnattereri	m	juv	0
60	2011	Brunnen Mey	Mnattereri	m	juv	0

60	2011	Brunnen Mey	Mnattereri	m	juv	0
60	2011	Brunnen Mey	Mnattereri	m	juv	0
60	2011	Brunnen Mey	Mnattereri	m	juv	1
60	2011	Brunnen Mey	Mnattereri	m	juv	1
60	2011	Brunnen Mey	Mnattereri	m	juv	1
60	2011	Brunnen Mey	Mnattereri	m	juv	3

smission opportunities in three European bat species.
ology, Eberhard-Gwinner-Strasse, 82319 Seewiesen, Germany
d, Germany.
